# Selection favors cost-ordered adaptive mutational paths in a stress-magnitude-dependent manner

**DOI:** 10.1038/s44319-026-00933-y

**Published:** 2026-09-12

**Authors:** Anjali V Patil, Jiao Zhao, Snehal V Khairnar, Sonali Srivastava, Laurence Yang, Amitesh Anand

**Affiliations:** 1 Department of Biological Sciences, Tata Institute of Fundamental Research, Mumbai, India; 2 Department of Chemical Engineering, Queen’s University, Kingston, Ontario Canada; 3 BRIC-National Institute of Immunology, Delhi, India

**Keywords:** Computational Biology, Evolution & Ecology, Membranes & Trafficking

## Abstract

Chronic exposure to stress requires adaptive strategies beyond canonical regulatory mechanisms. Stress varies, both qualitatively and quantitatively, across physiological niches and exerts distinct selection pressures on colonizing bacteria. Bacteria employ diverse defense strategies to withstand various stressful conditions, yet how they tailor their responses to different magnitudes of the same stressor remains poorly understood. We used multiple adaptive laboratory evolution experiments of *Escherichia coli* across varying paraquat concentrations and genetic backgrounds to dissect adaptive strategies at different levels of stress. Integrating multi-omic analyses with a tailored genome-scale metabolic model-based parametrized cost calculations, we identify two fundamentally distinct tolerance mechanisms. Under low-paraquat stress, blocking the polyamine transporter that is reported to be hijacked for paraquat influx suffices to maintain optimal growth. In contrast, higher stress levels activate an energetically demanding program involving enhanced detoxification and efflux. The transport flux regulation establishes a primary defense layer, upon which metabolic repair systems provide additional fitness advantages. The stress magnitude-dependent differential engagement of previously reported paraquat tolerance approaches offers insights into the principles governing dynamic bacterial adaptation.

## Synopsis

In *E. coli* import flux regulation provides tolerance to low paraquat stress while at higher paraquat stress, energetically demanding enhanced detoxification and efflux become critical, indicating that the mutational paths in response to stress levels are determined by cellular metabolic costs.

- Low paraquat stress is tolerated primarily by blocking the polyamine transporter based paraquat influx.
- High paraquat stress requires enhanced enzymatic detoxification and efflux, despite having higher energetic costs.
- Parametrized genome-scale metabolic model-based calculations demonstrate a cost-ordered selection of different adaptive responses.

## Abstract (web summary)

In *E. coli* import flux regulation provides tolerance to low paraquat stress while at higher paraquat stress, energetically demanding enhanced detoxification and efflux become critical, indicating that the mutational paths in response to stress levels are determined by cellular metabolic costs.

## Graphical abstract

**Figure:**
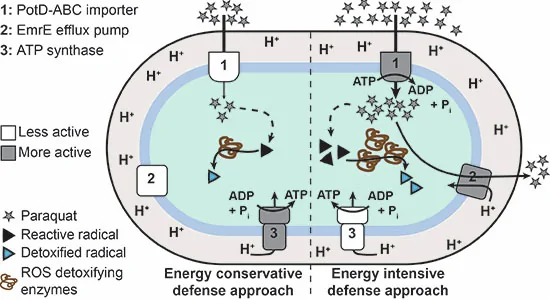

## Introduction

Stress mitigation is fundamental to life, and organisms have evolved diverse adaptive strategies to endure environmental perturbations (Taborsky et al, 2021). Bacteria are directly exposed to their environment, which is often dynamic and hostile. Bacteria, therefore, must survive a multitude of stresses like nutrient availability, pH, oxidative stress, and thermal stress, among other factors, to colonize various niches (Srivastav et al, 2025). Despite possessing a relatively simpler genome, bacterial stress response pathways are highly versatile in mitigating a wide array of stress conditions (Sharma et al, 2025; Dawan and Ahn, 2022; Chebotar’ et al, 2021). While the bacterial responses to distinct stresses are relatively well characterized, the mechanisms underlying adaptation to varying magnitudes of a stress remain poorly understood. This gap is especially relevant because various niches, such as distinct host tissues, often impose quantitatively varying stress and thereby exert differential selection pressures on colonizing populations (Elexpe et al, 2021; Tahara et al, 2009; Martínez, 2017).

Oxidative stress, arising from reactive oxygen species (ROS), represents one of the most pervasive threats encountered by bacteria, arising from diverse sources such as host immune responses, metabolic disturbances, and antibiotic exposure (Imlay, 2013). Bacteria are equipped with a suite of ROS defense pathways to orchestrate a tailored response to the specific nature of oxidative stress. The transcriptional regulators SoxR, OxyR, and RpoS can evoke specific transcriptional changes to restore redox homeostasis (Chiang and Schellhorn, 2012). In addition, chronic exposure to oxidative stress can lead to genetically programmed metabolic changes (Rychel et al, 2023; Anand et al, 2020). However, such genetically fixed adaptive changes often have a fitness trade-off associated with them (Anand et al, 2020). Therefore, careful resource allocation for growth versus defense systems governs optimal adaptive physiology.

We chose paraquat to impose different magnitudes of the same stress on *Escherichia coli*. The redox cycling chemistry of paraquat results in elevated oxygen consumption, depletion of NADPH, and generation of superoxide anion, resulting in diverse impacts on the biological system, including oxidative stress, biomolecular damage, and other cytotoxicities (Hassan and Fridovich, 1979; Alizadeh et al, 2022; Sullivan and Montgomery, 1986; Gray et al, 2007). We performed four separate adaptive laboratory evolutions (ALE) of *E. coli* under different paraquat concentrations and with varied genetic backgrounds to delineate adaptive strategies to distinct magnitudes of paraquat stress. Based on the multi-omic characterization of the evolved strains and tailored genome-scale model-based parametrized computations, we report two distinct paraquat stress-tolerance strategies in *E. coli* that are differentially engaged depending on the level of paraquat exposure. At low concentrations, limiting intracellular paraquat influx through a previously reported polyamine uptake system is sufficient for survival, whereas higher levels trigger an energetically costly program involving ROS detoxification and active efflux. The higher paraquat efflux was achieved through increased expression of EmrE, a multidrug efflux pump, which we earlier observed as a target for paraquat stress adaptation (Rychel et al, 2023). The transport flux control-based stress mitigation strategies allow further tolerance development using metabolic alterations. These findings highlight how cells modulate adaptive programs in response to the magnitude of stress, with implications for understanding the principles of dynamic stress-responsive regulation.

## Results

### Adaptive response of *E. coli* to varying paraquat levels

**Figure 1:**
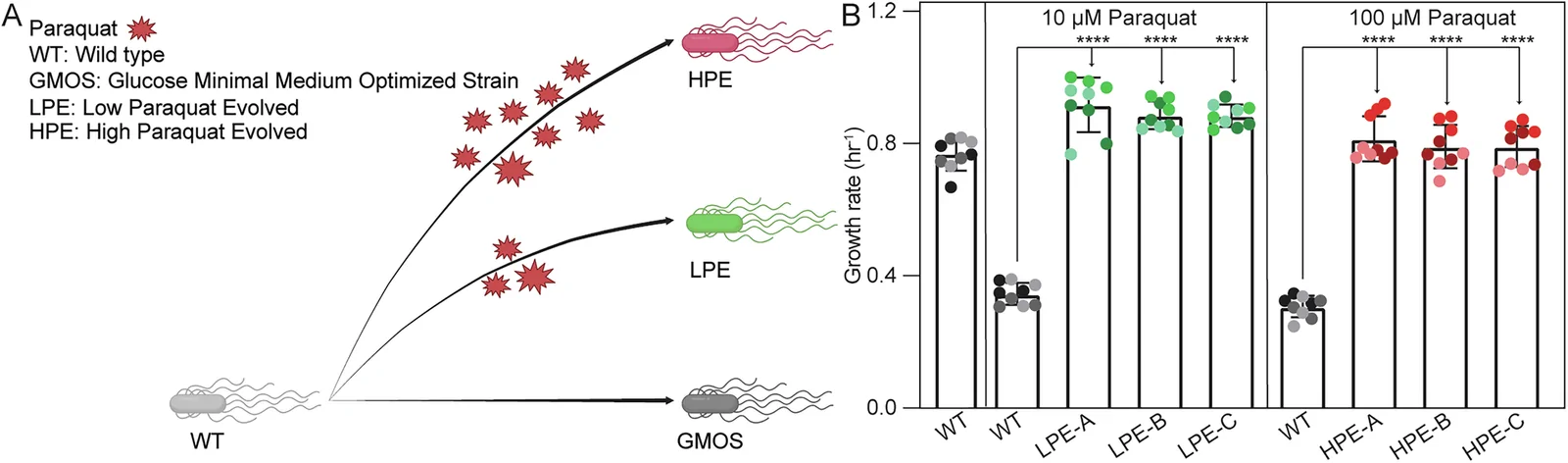
Adaptive evolution of *E. coli* in the presence of different levels of paraquat. (**A**) Schematic of the adaptive laboratory evolution (ALE) design. (**B**) Growth rates of *E. coli* before and after ALE in the presence of 10 μM and 100 μM paraquat. Bar graphs represent the mean of three biological replicates (each with three technical replicates (in the same color shade)), and the error bars show the standard deviation. Significance was determined using the Mann–Whitney test (****P value < 0.0001). Source data are available online for this figure.

Bacteria rely on transcriptional programs to maintain cellular homeostasis during transient fluctuations in stress levels (Donati et al, 2011; Imlay, 2013; Khademian and Imlay, 2021). However, sustained exposure to a selection pressure necessitates broader adaptive strategies, including lifestyle shifts and metabolic reprogramming (Imlay, 2013; Rychel et al, 2023). While there is a general understanding of multiple strategies associated with stress tolerance, the mechanistic distinction and stress level specialization are lacking (Rychel et al, 2023). To investigate mechanisms underlying long-term adaptations, we performed ALE of *E. coli* under two distinct paraquat concentrations differing by an order of magnitude (Fig. 1A). Three independent lineages were evolved in each condition to derive common adaptive traits, and ALE was continued until growth-rate improvements plateaued. Strains evolved at 10 µM paraquat are hereafter designated LPE-A/B/C (Low Paraquat Evolved), and those evolved at 100 µM paraquat are designated HPE-A/B/C (High Paraquat Evolved) (Appendix Table S1).

As expected, wild-type (WT) showed a significant growth rate drop at both paraquat concentrations (Fig. 1B). Evolved strains achieved significantly higher paraquat tolerance and recovered their growth rate (Fig. 1B). In paraquat-free medium, LPE and HPE showed growth profiles similar to a WT strain, GMOS, that is evolved to grow to its maximum growth rate in M9 minimal medium with glucose as a carbon source (Appendix Fig. S1) (LaCroix et al, 2015). In the presence of paraquat, while GMOS fails to grow, LPE and HPE show robust growth. Interestingly, the LPE strains are specialized for lower paraquat tolerance, as their growth was limited at higher levels of paraquat with an extended lag phase (Appendix Fig. S1). In contrast, HPE strains were tolerant to both paraquat concentrations. These phenotypic distinctions suggested a stress specialization and difference in underlying adaptive strategies between LPE and HPE.

### Distinct strategies to tolerate different magnitudes of paraquat stress

Stable adaptive phenotypic changes are often driven by genetic selection (Patil et al, 2024). We performed whole-genome sequencing of the evolved strains to compare their genotypes. Both LPE and HPE acquired a common set of mutations that are well characterized to improve the growth of *E. coli* on M9 minimal medium (Appendix Table S2). The mutations in RNA polymerase subunits, as well as in the intergenic region of *pyrE* and *rph*, genes facilitate growth-supporting cellular resource allocation (LaCroix et al, 2015; Conrad et al, 2010; Patil et al, 2024). This observation explains the growth similarity of LPE and HPE with GMOS in paraquat-free medium (Appendix Fig. S1). Remarkably, besides these common growth-promoting mutations, LPE and HPE strains showed mutations that were specific to stress levels.

**Figure 2:**
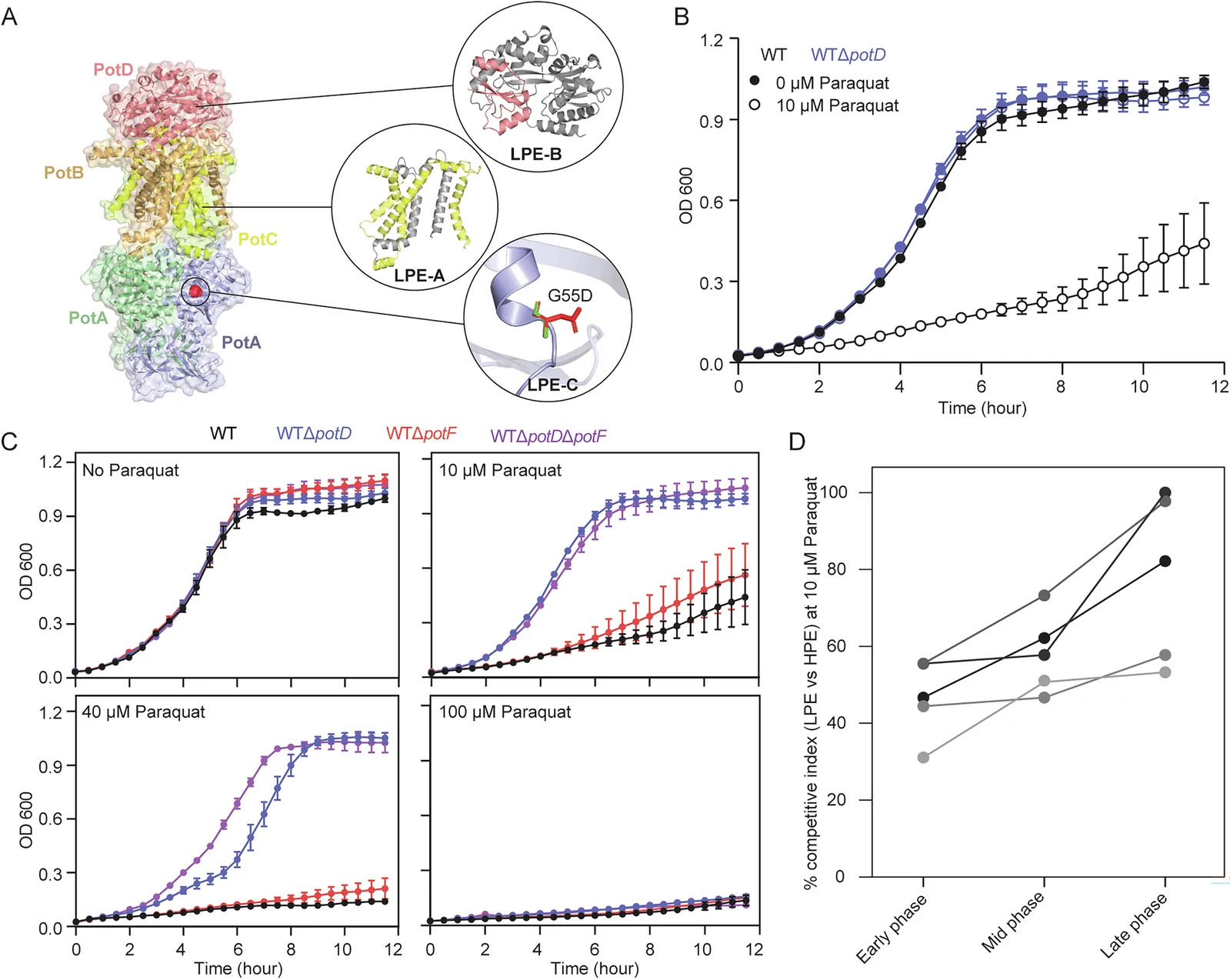
Adaptive strategy of paraquat stress tolerance in low-paraquat evolved *E. coli*. (**A**) The mutations observed in the LPE strains mapped on the PotD-PotABC complex from *E. coli* (Structure of PDB ID 8Y5I rendered using PyMol). In the zoomed-in insets of PotD and PotC, the regions shown in gray depict the truncated region. In the zoomed-in inset of PotA, green represents a wild-type amino acid residue, while red represents a mutant amino acid residue. (**B**) Effect of *potD* gene deletion on the growth profile of the WT strain in the presence of 10 μM paraquat. The growth curve shows a mean of four biological replicates (with three technical replicates each), and the error bars show the standard error of the mean. (**C**) Effect of *potD* and *potF* gene deletion on the growth profile of the WT strain in the presence of different paraquat concentrations. The growth curve shows a mean of four biological replicates (with three technical replicates each), and the error bars show the standard error of the mean. (**D**) Percent competitive index of WTΔ*potD* (an allele-level proxy for LPE) vs HPE in the presence of 10 μM paraquat. All five individual biological replicates are shown on the plot. Source data are available online for this figure.

All three independently evolved lineages of LPE acquired mutations in an ABC transporter PotD-PotABC. While this transporter is responsible for the uptake of polyamine, the structural analogy can enable cellular entry of paraquat through this system. Putrescine uptake decreases in the presence of paraquat, suggesting competition for the same transporter (Kashiwagi et al, 1990). Consistently, paraquat toxicity is mitigated by the presence of polyamines in the medium, with spermidine being more effective than putrescine (Minton et al, 1990). It has been hypothesized that, while some paraquat enters passively, the active transport takes place by an energy-dependent transport mechanism (Kao and Hassan, 1985). LPE-A and LPE-B acquired mutations in *potC* and *potD*, respectively, that truncated the open reading frames (Fig. 2A). In contrast, a point mutation in LPE-C results in a PotA G55D substitution within its ATP-binding domain. This substitution introduces a negatively charged side chain and alters the residue’s helical propensity, which may disrupt protein folding (Lyu, 1991; O’Neil and DeGrado, 1990). Such mutations should result in loss of function of this transporter and potentially limit paraquat influx. We generated a *potD* knockout strain to establish the role of PotD-PotABC disruption in paraquat tolerance. WT∆*potD* displayed comparable growth to the WT strain under non-stress conditions but exhibited improved tolerance to 10 μM paraquat (Fig. 2B). These results suggest that inactivation of the PotD-PotABC transporter confers a growth advantage under paraquat stress by limiting the non-specific paraquat entry into the cell.

*E. coli* also encodes a second polyamine uptake system, PotF-PotGHI, which is specific for putrescine (Igarashi et al, 2001); however, this pathway was not selected during growth optimization under paraquat stress. To examine the basis of this apparent evolutionary bias, we compared paraquat tolerance in WTΔ*potD* and WTΔ*potF* strains (Fig. 2C). In contrast to *potD* deletion, loss of *potF* alone did not confer an appreciable tolerance at 10 μM paraquat. Notably, simultaneous deletion of *potF* and *potD* further enhanced tolerance relative to WTΔ*potD*. These results explain the preferential selection of the PotD-PotABC system and indicate that PotF-PotGHI does not serve as the primary route for paraquat influx. This observation of import route preference is consistent with the report of higher paraquat tolerance achieved by spermidine supplementation than putrescine (Minton et al, 1990). An important question emerging from this observation is whether the distinct substrate specificities for individual polyamine species influence their differential contribution to paraquat uptake. Notably, this import restriction-based strategy provides limited tolerance, and at higher paraquat concentrations, the strains were significantly susceptible (Appendix Fig. S1). However, the adaptive strategy of LPE seems to be better suited for lower paraquat concentrations than HPE. We performed competitive growth profiling by co-inoculating WTΔ*potD* (an allele-level proxy for LPE) and HPE in a medium containing 10 μM paraquat. We observed a progressive increase in the representation of LPE cells over time, indicating the superiority of the tolerance mechanism of LPE at this concentration (Fig. 2D).

**Figure 3:**
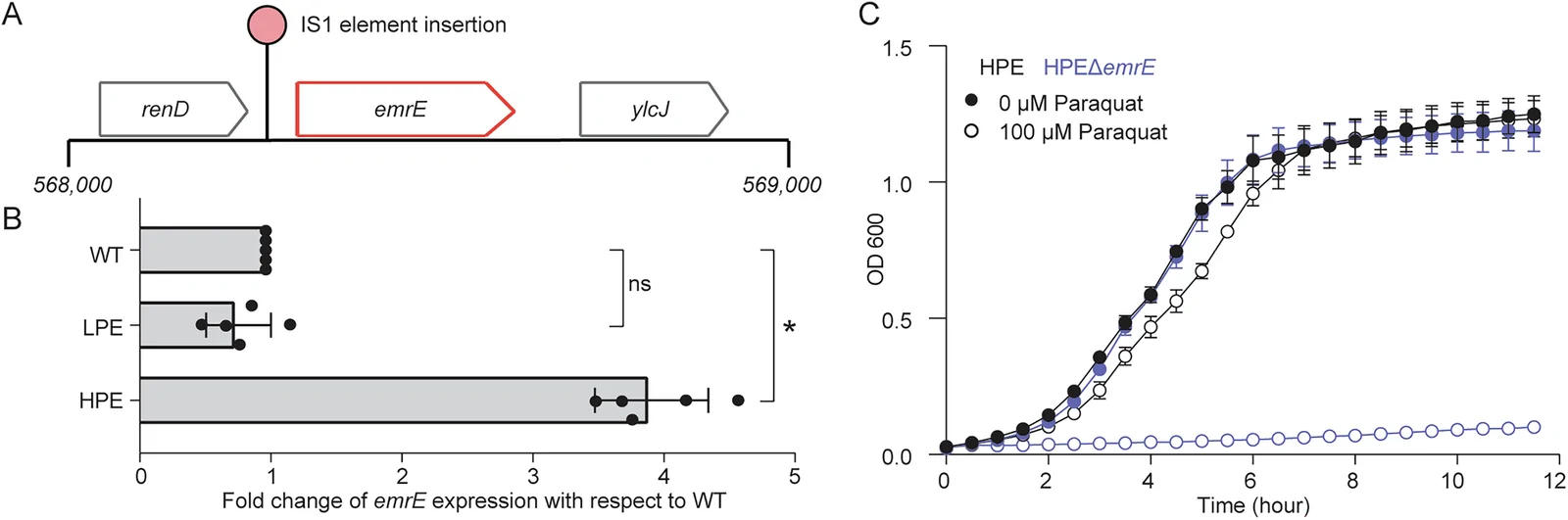
Adaptive strategy of paraquat stress tolerance in high-paraquat evolved *E. coli*. (**A**) Schematic for the mutation observed in the HPE strains. (**B**) Relative expression of the *emrE* gene in LPE and HPE strains with respect to the WT. All five replicate values are displayed. Bar graphs represent the mean, and error bars represent the standard deviation. Significance was determined using the Kruskal–Wallis test (*P value = 0.0308). (**C**) Effect of *emrE* gene deletion on the growth profile of HPE strain in the presence of 100 μM paraquat. The growth curve shows a mean of three biological replicates (with three technical replicates each), and the error bars show the standard error of the mean. Source data are available online for this figure.

We then examined the genetic changes in HPE lineages. Interestingly, none of the strains evolved at higher paraquat concentration showed polyamine transporter-related mutations. Rather, all three lineages of HPE acquired an insertion element, IS1, upstream of the *emrE* gene (Fig. 3A). An adaptive targeting of this region in the form of a similar insertion or a large amplification was observed in response to paraquat toxicity (Rychel et al, 2023). Since such insertions can impact the expression levels, we examined the expression of *emrE* in evolved strains as compared to WT (Grindley, 1978; Fahrner and Berg, 2015; Rychel et al, 2023). We observed a significantly higher expression of *emrE* in HPE, consistent with the previously reported iModulon activity (Fig. 3B). The gene *emrE* encodes a multidrug efflux pump that is implicated in paraquat export (Morimyo et al, 1992; Yerushalmi et al, 1995). The deletion of *emrE* in HPE resulted in a complete loss of paraquat tolerance (Fig. 3C). These observations suggest functionally opposite transport flux control strategies for paraquat tolerance and growth optimization in HPE as compared to LPE. This stress magnitude-dependent resolution of paraquat-tolerizing mutational paths motivated us to examine the distinction in underlying metabolic optimization.

### Metabolic influence of different tolerance strategies

**Figure 4:**
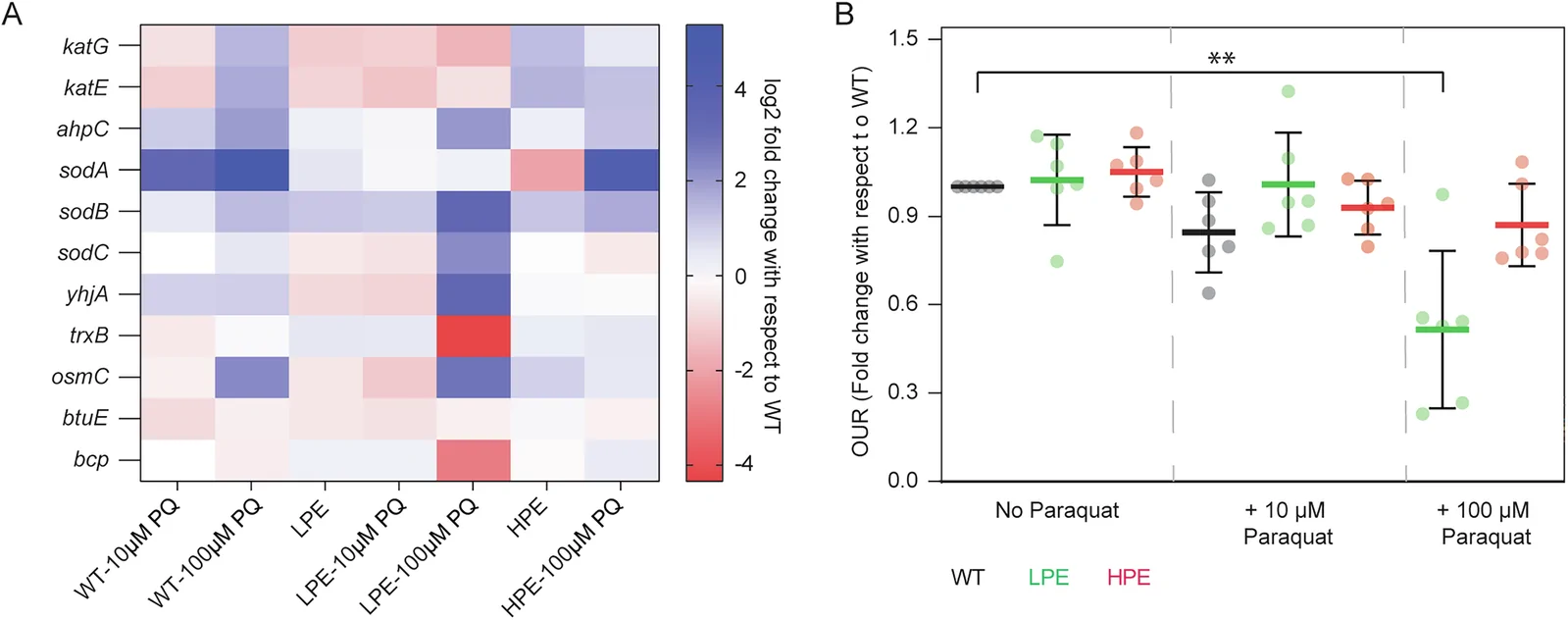
Transcriptional and respiratory responses to paraquat exposure. (**A**) Heatmap showing expression levels (log2 fold change with respect to WT) of the ROS mitigation-related genes when treated with paraquat (PQ). The data are an average of three biological replicates. (**B**) Oxygen uptake rates of the strains in the presence and absence of the specified paraquat concentrations. All six individual biological replicates are shown on the plot. The mean is represented by the bold solid lines, and the error bars represent the standard deviation. Significance was determined using the Kruskal–Wallis test, and except for LPE-100 μM Paraquat (which showed ** significance), all others showed non-significance in comparison to WT-No Paraquat. (**P value = 0.0032). Source data are available online for this figure.

The specialization in the tolerance strategies motivated us to examine the physiological logic behind the distinct evolutionary preference. Given that paraquat is known to generate superoxide radicals, we started by examining the expression status of the genes encoding canonical ROS detoxifying enzymes (Fig. 4A) (Hassan and Fridovich, 1979; Donati et al, 2011). WT showed an expected paraquat dose-dependent upregulation of superoxide dismutases and other enzymes. LPE strains did not upregulate these enzymes at 10 μM paraquat concentration, likely due to reduced influx of paraquat and hence lesser stress. However, at higher paraquat concentration, LPE is required to upregulate ROS-mitigating enzymes, suggesting limited transport flux-restriction-based tolerance. In contrast, HPE strains showed higher expression of these enzymes and are likely using both enzymatic detoxification and elevated paraquat efflux to maintain redox homeostasis.

Aerobic respiration is the primary source of ROS, and paraquat stress is reported to move *E. coli* to a lower aerotype wherein energy dependency on the electron transport system is reduced (Rychel et al, 2023; Messner and Imlay, 1999). We estimated the oxygen uptake rates of strains with various paraquat concentrations (Fig. 4B). We did observe a reduction in oxygen consumption in LPE at higher levels of paraquat, a potential strategy to reduce ROS generation. However, the higher paraquat tolerance of HPE strains allowed them to avoid this metabolic downshift. Thus, the LPE and HPE strains seem to be engaging two different metabolic programs to deal with their respective redox stress.

### Cost associated with deploying different tolerance strategies

Our findings show that potentially LPE decreases paraquat uptake, whereas HPE enhances its export alongside increased ROS detoxification. Although both approaches successfully alleviate paraquat stress, the reason for the preference of one strategy over the other under conditions of persistently low versus high stress is not clear. To investigate this, we employed a computational analysis to determine the relative costs of each strategy, motivated by the principle that bacterial metabolic choices are often governed by resource allocation constraints (Basan, 2018; Dalldorf et al, 2024).

For this purpose, we used the StressME model, a unified genome-scale model of *E. coli* metabolism, gene expression, and stress responses that can compute complete metabolic network rewiring under various stresses (Zhao et al, 2024). The model accounts for the cellular costs of transport: the proton motive force (PMF) consumed by EmrE and the ATP used to operate PotD-PotABC. Consequently, the energy consumption (in the form of PMF or ATP) of both transporters can be simulated according to the adaptation strategy (LPE vs. HPE).

**Figure 5:**
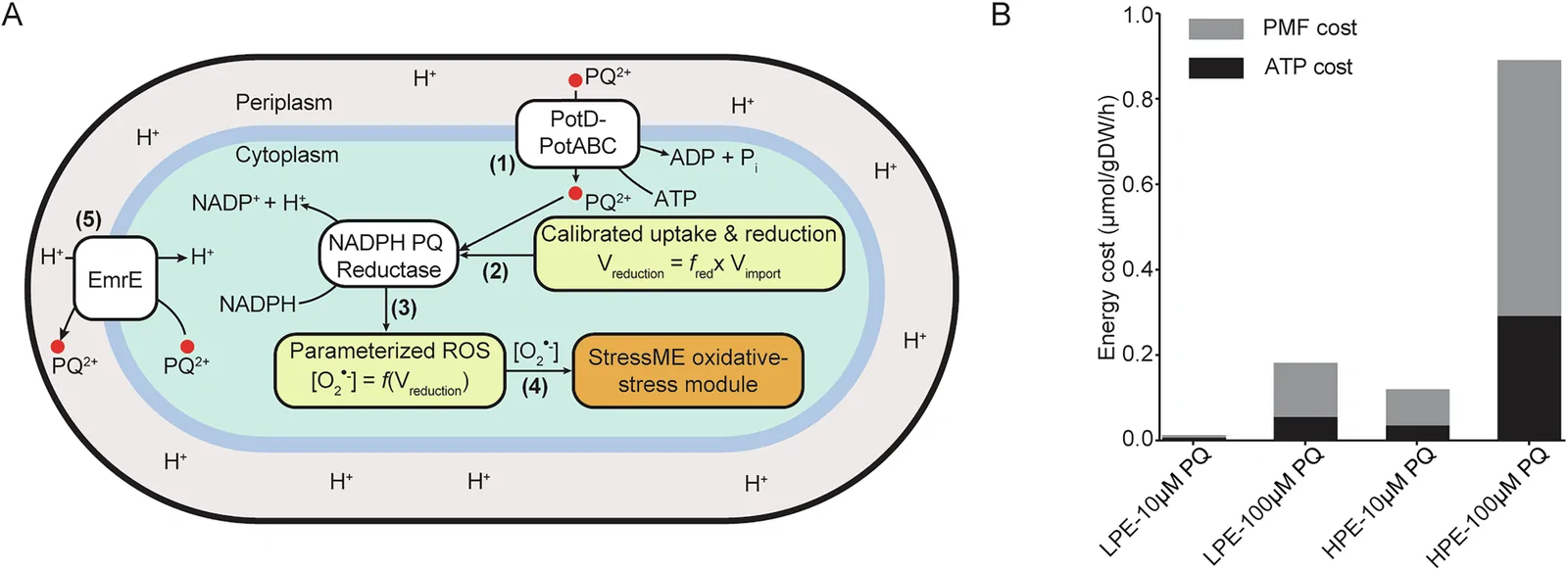
Estimation of energetic costs associated with different magnitudes of paraquat stress. (**A**) Parameterized implementation of paraquat transport and redox cycling pathways in StressME. (**B**) Genome-scale model-based energy cost (PMF and ATP) estimates of the paraquat evolved strains when exposed to 10 μM and 100 μM paraquat. Source data are available online for this figure.

We incorporated the paraquat transport and redox-cycling pathway into StressME by extending the model with paraquat transport reactions and a parameterized intracellular redox-cycling module (Fig. 5A; “Methods”; Appendix Table S3). Specifically, PotD-PotABC-mediated paraquat import (1) and EmrE-mediated paraquat export (5) were represented as explicit ME reactions, whereas the intracellular paraquat reduction flux (2) was parameterized using the calibrated intracellular cycling parameter (*f*red), and the resulting intracellular superoxide burden (3) was parameterized as a function of the reduction flux. The parameterized superoxide burden was subsequently propagated through the StressME oxidative-stress framework (4) to predict ROS detoxification, repair of damaged cellular components, proteome allocation, energetic costs, and growth. This implementation enables simultaneous accounting of energetic costs, protein investment, and redox consequences associated with alternative detoxification strategies. Using the updated model, we quantified PMF and ATP demands associated with paraquat handling under strain-specific optimal split ratios and extracellular paraquat concentrations, enabling direct comparison of the energetic burden imposed by the adaptive strategies of the LPE and HPE strains.

The simulation predicts a difference in PMF and ATP requirements between LPE and HPE strains. At both 10 μM and 100 μM paraquat concentrations, LPE appears to invest lesser energy resources as compared to HPE (Fig. 5B). These cost estimations suggest that, while effective at high stress, the genetically fixed, high-energy adaptive approach of HPE is disadvantageous at lower stress levels. This potential difference in energy demand further explains the observation of LPE outcompeting HPE at 10 μM paraquat concentration (Fig. 2D) and the logic behind the evolutionary selection of an entry-restriction-based strategy for the lower paraquat evolution paradigm.

### Tolerance upshift in evolved strains

**Figure 6:**
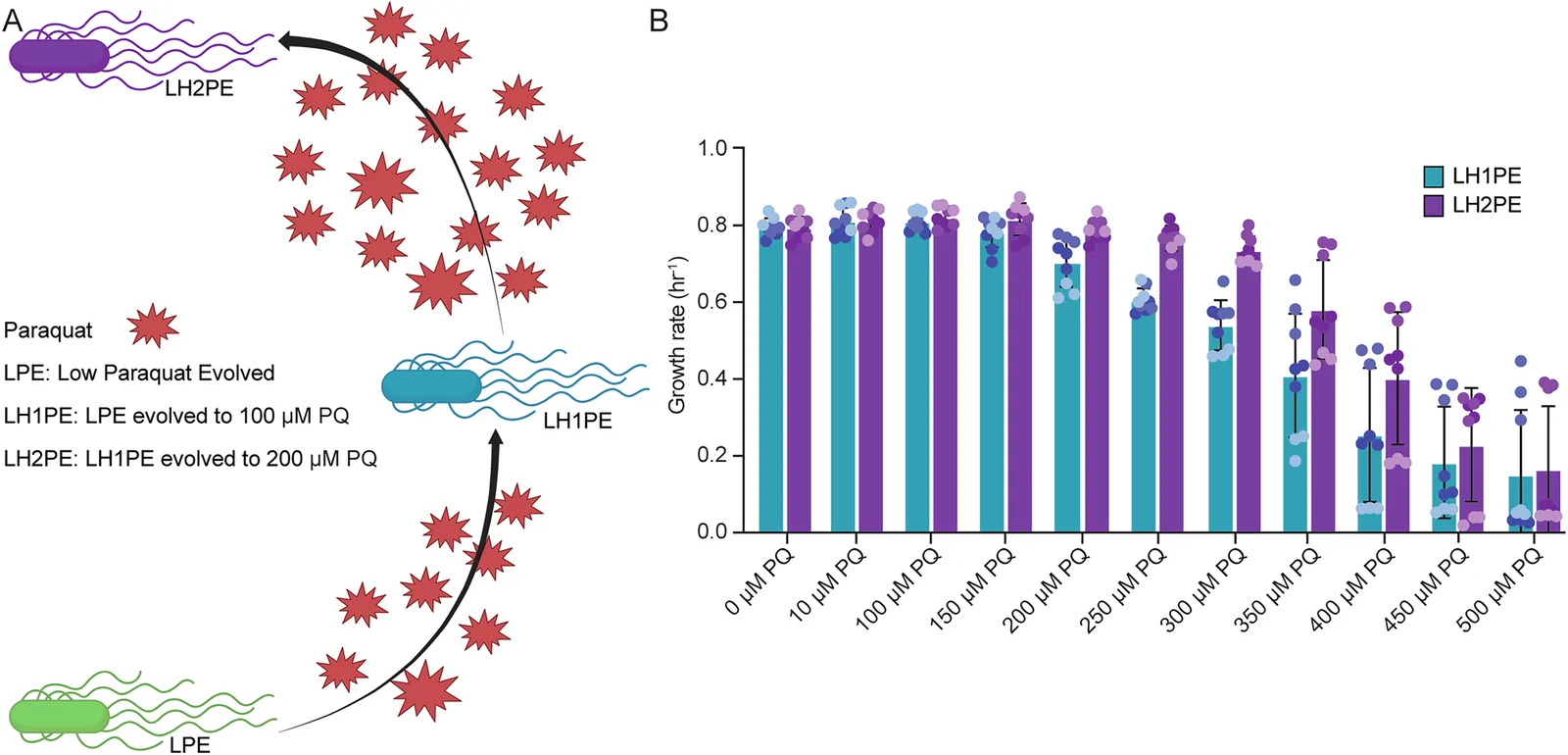
Adaptive evolution of LPE in the presence of higher levels of paraquat. (**A**) Schematic of the adaptive laboratory evolution (ALE) design. (**B**) Growth rates of the evolved strains upon exposure to different paraquat (PQ) concentrations. Bar graphs represent the mean of three biological replicates (each with three technical replicates (in the same color shade)), and the error bars show the standard deviation. Source data are available online for this figure.

The two distinct adaptive mechanisms specific to different ranges of paraquat motivated us to delineate additional layers of tolerance strategies in *E. coli*. Towards this objective, we designed a series of ALE experiments (Fig. 6A). To elevate the stress tolerance, we evolved LPE strain under 100 μM paraquat stress. All three lineages of the LPE strain evolved to 100 μM paraquat (designated as LH1PE) acquired the earlier described efflux strategy, similar to HPE strains, to improve their tolerance (Appendix Table S2). However, the LH1PE strain, when further evolved at a doubled paraquat concentration (200 μM; designated as LH2PE), recovered mutations in the *sdhE*-*ygfZ* locus consistent with prior paraquat ALE studies (Rychel et al, 2023; Phaneuf et al, 2024).

YgfZ encodes an uncharacterized folate-dependent protein; however, its mitochondrial homolog, IBA57, is involved in the maturation of the Fe-S cluster (Gelling et al, 2008; Mühlenhoff et al, 2022). Given paraquat’s potential to generate oxidative stress and ROS sensitivity of Fe-S clusters, we examined whether the mutational targeting of *ygfZ* is directed to achieve additional tolerance (Imlay, 2006). We performed a comparative sensitivity profiling of LH1PE and LH2PE using a range of paraquat concentrations (Fig. 6B). Both strains showed similar tolerance up to 100 μM paraquat, but beyond this concentration, LH2PE started to show a clear dose-dependent tolerance superiority. Notably, while two lineages of LH2PE showed mutations in the gene, one lineage selected for an intergenic mutation between *sdhE* and *ygfZ*. Despite the selection bias towards *ygfZ*, the potential adaptive targeting of *sdhE* in the LH2PE-B for paraquat tolerance cannot be ruled out, given the ROS sensitivity of the Fe-S cluster of SdhABCD and involvement of SdhE in its flavinylation (McNeil et al, 2012). This observation highlights the metabolic augmentation of ROS defense built upon transport flux control strategies.

## Discussion

Adaptive evolution is often viewed as an accumulation of beneficial mutations that increase stress tolerance. Our results suggest that adaptation to chronic paraquat exposure is governed by selection among multiple accessible evolutionary trajectories, whose fitness depends on the magnitude of the imposed stress rather than on stress alone. Across independent evolutionary lineages, *E. coli* repeatedly converged on distinct transport-regulation-centered solutions that minimized the physiological burden of paraquat toxicity, revealing that stable adaptation is fundamentally a resource-allocation problem in which metabolic economy determines which mutational paths are favored.

The sequential evolution experiments recovered several adaptive mutations reported for paraquat tolerance and ordered them as specific stress magnitude responses. This observation highlights a critical gap in generic laboratory evolution experiments designed for studying adaptive responses to stress, which is likely to identify only a subset of the physiological solutions potentially available to a population.

Although both LPE and HPE achieve improved paraquat tolerance, genome-scale modeling predicts substantially lower energetic investment for the transport-restriction strategy than for the combined efflux-detoxification approach. This prediction is consistent with the competitive fitness advantage of the LPE strategy under low-paraquat concentrations and suggests that evolutionary selection optimizes long-term resource expenditure rather than only maximizing stress resistance. Importantly, these results emphasize that the fitness of an adaptive mutation cannot be evaluated independently of its physiological cost and the environmental context in which it operates. Resource allocation therefore represents an important constraint shaping evolutionary outcomes.

Such graded deployment of defense strategies may represent a widespread principle extending beyond paraquat exposure to other antimicrobial, metabolic, and host-derived stresses. Because many clinically relevant environments expose bacteria to persistent but heterogeneous stress levels, understanding how stress magnitude shapes evolutionary trajectories may improve our ability to predict resistance evolution and identify vulnerabilities that emerge from the physiological compromises accompanying adaptation.

## Methods

**Table:** Reagents and tools table

Reagent/resource	Reference or source	Identifier or catalog number
**Experimental models**		
*Escherichia coli* K12 MG1655	ATCC	700926
**Oligonucleotides and other sequence-based reagents**		
*K1 :* CAGTCATAGCCGAATAGCCT	Sigma-Aldrich	Customized
*potD* forward primer: GACGATGTGGTAGTTTCTTC	Sigma-Aldrich	Customized
*potD* reverse primer: ACGGTATCAACAATGACGCC	Sigma-Aldrich	Customized
*potF* forward primer: TACCGTTTTTTAATCCGAGC	Sigma-Aldrich	Customized
*potF* reverse primer: ACCATCGTAGGATTTGGTCA	Sigma-Aldrich	Customized
*emrE* forward primer: AGCACGAATCTGATTTTTAA	Sigma-Aldrich	Customized
*emrE* reverse primer: CAATTAAGGTCACATTATCT	Sigma-Aldrich	Customized
*emrE* qPCR forward primer: TCTGGTCAGGAGTCGGTATT	Sigma-Aldrich	Customized
*emrE* qPCR reverse primer: CACCGGCACAAATCAACATC	Sigma-Aldrich	Customized
*rpoD* qPCR forward primer (house-keeping): GAGCAAGGCTATCTGACCTATG	Sigma-Aldrich	Customized
*rpoD* qPCR reverse primer (house-keeping): GCCCATGTCGTTGATCATTTG	Sigma-Aldrich	Customized
**Chemicals, enzymes, and other reagents**		
CaCl_2_.2H_2_O	Sigma-Aldrich	C3306
MgSO_4._7H_2_O	SRL	1344116
Na_2_HPO_4_	Sigma-Aldrich	S9763
KH_2_PO_4_	Sigma-Aldrich	P5655
NaCl	MP Bio	194848
NH_4_Cl	Sigma-Aldrich	A4515
FeCl_3_.6H_2_O	Sigma-Aldrich	F2877
ZnCl_2_ anhydrous	Sigma-Aldrich	Z1052
CoCl_2_.6H_2_O	Sigma-Aldrich	C8661
Na_2_MoO_4_.2H_2_O	Sigma-Aldrich	M1651
CuCl_2._2H_2_O	Sigma-Aldrich	C3279
H_3_BO_3_	USB	76324
Concentrated HCl	Sigma-Aldrich	H1758
Glucose	Sigma-Aldrich	49139
Paraquat	Sigma-Aldrich	36541
Kanamycin	Sigma-Aldrich	K1377
Luria–Bertani agar	Himedia	M1151
TRIzol reagent	Thermo Fisher Scientific	15596018
Chloroform	SDFCL	3771OUR
Absolute ethanol	Honeywell	32221
X-Gal	Sigma-Aldrich	10745740001
QIAamp PowerFecal Pro DNA Kit	Qiagen	51804
QIAseq® FX DNA library kit	Qiagen	180477
QIAseq FastSelect –5S/16S/23S Kit	Qiagen	335925
First Strand cDNA synthesis kit	Thermo Scientific	18091050
SsoAdvanced Universal SYBR Green Supermix	Bio-Rad	1725270
**Software**		
GraphPad Prism	Version 10.0.2	Graph pad.com
gcplyr	https://github.com/mikeblazanin/gcplyr	N/A
PyMol	Version 3.0	N/A

### Bacterial strains and growth conditions

*Escherichia coli* K12 MG1655 (ATCC 700926) was used as the wild-type strain. All the growth assays were performed using the Tecan Spark multimode microplate reader at 37 °C and atmospheric oxygen partial pressure using a 96-well plate with 200 μL culture per well. At least three biological replicates (each with three technical replicates) were performed for each experiment. Unless stated otherwise, M9 minimal medium supplemented with 4 g/L glucose was used for bacterial growth. For the growth profiling with media variation, paraquat was added to M9 minimal medium supplemented with 4 g/L glucose at the required concentrations.

*potD, potF*, and *emrE* knockout strains were generated using the P1 phage transduction method, with Keio collection strains as donors for gene knockout cassettes (Thomason et al, 2007; Baba et al, 2006).

Growth rate calculations were done using gcplyr: an R package for microbial growth curve data analysis (Blazanin, 2024).

### Adaptive laboratory evolution

Adaptive laboratory evolution was performed using three independent replicates of the *E. coli* wild-type strain. Low-paraquat evolution was performed by serially propagating the cultures in a 7 mL M9 minimal medium supplemented with 4 g/L glucose and 10 μM paraquat at 37 °C and atmospheric oxygen partial pressure. Once they had reached mid-exponential phase (OD_600_ ~ 0.6–1), the cultures were propagated in the fresh medium and were set to a starting OD_600_ of 0.1. Cultures were always maintained in excess nutrient conditions, assessed by non-tapering exponential growth. The laboratory evolution was performed for a sufficient interval to allow the cells to reach their growth rate plateau. The high-paraquat evolution was performed in the same conditions with 100 μM paraquat. Further evolution of LPE in the presence of 100 μM paraquat and LH1PE in the presence of 200 μM paraquat was performed in a similar manner.

The clones from the endpoints of each evolved lineage were picked by streaking the cultures on Luria–Bertani agar plates and were used as representatives for all the experiments. (Appendix Table S1).

### DNA resequencing

DNA resequencing was performed on clones from the endpoints of evolved strains. Total DNA was sampled from an overnight culture and immediately centrifuged for 5 min at 12,000 r.p.m. The supernatant was decanted, and the cell pellet was frozen at −80 °C. Genomic DNA was isolated using QIAamp PowerFecal Pro DNA Kit (Cat No./ID: 51804, QIAGEN GmbH) following the manufacturer’s protocol. Resequencing libraries were prepared using QIAseq® FX DNA library kit from Qiagen, following the manufacturer’s protocol. Libraries were run on a HiSeq and/or NextSeq (Illumina).

Sequencing reads were filtered and trimmed using the FastP software. The breseq bioinformatics pipeline version 0.31.1 was used to map sequencing reads and identify mutations relative to an *E. coli* K-12 MG1655 reference genome (NC_000913.3) amended to reflect the starting strain best.

### Structure rendering and visualization in PyMol

Protein structure of PotD-PotABC (PDB ID: 8Y5I) of *E. coli* K-12 was acquired from the Protein Data Bank (PDB). The structure was rendered using PyMol to facilitate detailed visualization.

### RNA isolation, qPCR, and RNA sequencing

Bacteria were grown in M9 minimal medium supplemented with 4 g/L glucose, with respective paraquat concentration, till their mid-exponential phase. The culture was harvested and immediately processed for RNA isolation. The cells were dissolved in TRIzol and incubated at 65 °C for 15 min. RNA was obtained in an aqueous layer upon the addition of chloroform, which was then precipitated using chilled isopropanol. This RNA pellet was washed with alcohol and finally resuspended in RNase-free water. cDNA synthesis was performed using Thermo Scientific RevertAid First Strand cDNA synthesis kit using random hexamer primer, according to the manufacturer’s protocol. Quantitative real-time PCR was carried out with BioRad SsoAdvanced Universal SYBR Green Supermix in the CFX Opus 96 real-time PCR system (Bio-Rad).

The same RNA samples were used further for RNA sequencing. Total RNA concentration was measured using the Qubit RNA High Sensitivity assay according to the manufacturer’s instructions, and RNA integrity and fragment size distribution were evaluated using the Agilent Fragment Analyzer with a high-sensitivity RNA kit. RNA sequencing libraries were prepared using the QIAseq FastSelect rRNA depletion system (5S/16S/23S) in combination with the QIAseq RNA library preparation enzymes and buffers, following the manufacturer’s protocols. After library construction, final library concentrations were quantified using the Qubit dsDNA High Sensitivity assay, and library size distribution and quality were assessed with the Agilent high-sensitivity NGS fragment analysis system. Libraries were normalized and pooled at equimolar concentrations prior to sequencing, and the pooled libraries were sequenced on an Illumina NextSeq 2000 platform. Further analysis was performed as described in a previous study (Anand et al, 2019).

### Phenotype characterization

Culture density was measured at 600 nm absorbance with a spectrophotometer and correlated to cell biomass. Samples for exometabolite estimation were filtered through a 0.22-μm filter (PVDF, Millipore) and measured using refractive index detection by HPLC (Agilent 12600 Infinity) with a Bio-Rad Aminex HPX87-H ion exclusion column. The HPLC method was the following: injection volume of 10 μL and 5 mM H_2_SO_4_ mobile phase set to a flow rate and temperature of 0.5 mL/min and 45 °C, respectively, for a run time of 20 min per sample.

The oxygen uptake of each culture was determined by measuring the depletion of dissolved oxygen using the Oroboros O2K system. The cultures were grown in M9 minimal medium supplemented with 4 g/L glucose, with respective paraquat concentration, at 37 °C and atmospheric oxygen partial pressure, and oxygen uptake rates were normalized to OD_600_.

### Competition assay and relative fitness estimation

The relative fitness of paraquat-tolerant strains was assessed using pairwise competition assays. Log-phase monocultures of the WT∆*potD* strain (an allele-level proxy for LPE) and the HPE strain (lineage A) were mixed in a 1:1 ratio and inoculated into 15 mL of M9 minimal medium supplemented with 4 g/L glucose and 10 μM paraquat.

The mixed culture was incubated at 37 °C and an atmospheric oxygen partial pressure to allow competition between the strains. Samples were collected at three defined time points: early (0.5 h), mid (2 h), and late (7 h). At each time point, cultures were serially diluted and plated onto Luria–Bertani (LB) agar plates to determine total viable counts.

To distinguish WT∆*potD* from the competing population, individual colonies from LB plates were patch-plated onto LB agar containing kanamycin. Colonies that grew on LB-kanamycin plates were scored as WT∆*potD*. The competitive index was calculated as the proportion of WT∆*potD* colonies within the total population using the following formula:

$$\% \,{competitive\; index}=\frac{{No}.{of\; WT}\Delta {potD\; colonies\; growing\; on\; LB}-{Kan\; plates}}{{Total\; no}.{of\; colonies\; patched}}\,x\,100$$

### Simulation of paraquat transport and redox cycling

The biological paraquat transport and redox-cycling mechanism comprises four coupled processes (Eqs. (1)–(4)), including paraquat import, intracellular reduction, re-oxidation with superoxide generation, and paraquat export. In the computational implementation, the transport processes were represented explicitly in StressME, whereas the intracellular redox cycling was represented through calibrated parameterized relationships, as illustrated in Fig. 5A (Appendix Table S3).

1 $${{{\rm{pq}}}2}_{{{\rm{periplasm}}}}{{\rightarrow}^{{PotD}-{PotABC}}}{{{\rm{pq}}}2}_{{{\rm{cytoplasm}}}}$$

2 $${{{\rm{pq}}}2}_{{{\rm{cytoplasm}}}}+{{\rm{NADPH}}}\mathop{\longrightarrow }^{{{\rm{PQ}}}2{{\rm{RED}}}}{{{\rm{pq}}}1}_{{{\rm{cytoplasm}}}}+{{{\rm{NADP}}}}^{+}+{{{\rm{H}}}}^{+}$$

3 $${{{\rm{pq}}}1}_{{{\rm{cytoplasm}}}}+{{{\rm{O}}}}_{2}{{\longrightarrow }^{{{\rm{PQ}}}1{{\rm{OX}}}}}{{{\rm{pq}}}2}_{{{\rm{cytoplasm}}}}+\cdot {{{{\rm{O}}}}_{2}}^{\!\!-}$$

4 $${{{\rm{pq}}}2}_{{{\rm{cytoplasm}}}}{{\longrightarrow }^{{EmrE}}}{{{\rm{pq}}}2}_{{{\rm{periplasm}}}}$$

In the computational implementation, the paraquat import flux (${v}_{{import}}$) was computed from extracellular paraquat concentration using a Michaelis–Menten relationship (Eq. (5)):

5 $${v}_{{import}}=\frac{{V}_{max }[{pq}2]_{{ext}}}{{K}_{m}+[{pq}2]_{{ext}}}$$

where ${V}_{max }$ and ${K}_{m}$ were calibrated against experimental growth responses measured across a series of extracellular paraquat concentrations.

Rather than explicitly solving the intracellular redox cycle, the paraquat reduction flux was parameterized as

$${v}_{{reduction}}={{frac}}_{{red}}\,{v}_{{import}}$$

where ${{frac}}_{{red}}$, the flux split ratio for paraquat reduction, was calibrated separately for each strain against the experimental growth-response data.

The intracellular paraquat species satisfy the following mass-balance relationship:

6 $${v}_{{import}}={v}_{{reduction}}+{v}_{{export}}-{v}_{{oxidation}}$$

Under the assumption of steady state paraquat redox cycling, the reduction and re-oxidation fluxes are equal:

7 $${v}_{{oxidation}}={v}_{{reduction}}$$

which simplifies Eq. (6) to

8 $${v}_{{export}}={v}_{{import}}$$

The intracellular superoxide burden was subsequently parameterized as a function of the paraquat reduction flux:

9 $$\left[{{{\rm{O}}}}_{2}^{\cdot -}\right]=f\left({v}_{{{\rm{reduction}}}}\right)=\frac{{v}_{{{\rm{reduction}}}}}{{k}_{{{\rm{ROS}}}}}$$

where k_ROS_ is a calibrated conversion coefficient relating the paraquat reduction flux to the intracellular superoxide burden supplied to the StressME oxidative-stress module. The StressME framework subsequently predicts ROS detoxification, oxidative-damage repair, proteome allocation, energetic costs, and cellular growth.

Protein abundance ratios between PQ2RED and EmrE ([*P*_red_]/[*P*_EmrE_]) were approximated from RNA-seq transcript abundances assuming similar translation efficiencies:

10 $${prot}{{\_}}{ratio}={tans}{{\_}}{ratio}=\frac{[{mRN}{A}_{{red}}]}{[{mRN}{A}_{{EmrE}}]}$$

The effective catalytic rate constant for EmrE was then estimated as

11 $${k}_{{eff},{EmrE}}=\frac{{k}_{{eff},{red}}}{{{frac}}_{{red}}}\times {prot}{{\_}}{ratio}$$

The computed ${v}_{{reduction}}$ and ${v}_{{export}}$ were subsequently used to constrain the corresponding PQ2RED and EmrE reactions within the StressME model. Consequently, the parameterized paraquat transport and intracellular redox-cycling module established the strain-specific ROS burden, whereas the downstream oxidative-stress responses, including detoxification, repair, proteome allocation, transport energetics, and growth, were predicted by the StressME framework. (Appendix Table S4 summarizes the role of each model quantity, distinguishing calibrated parameters, experimental inputs, intermediate calculations, and ME model predictions).

### Cost-benefit analysis of EmrE-mediated paraquat export

To quantify the cellular cost and benefit of EmrE-mediated paraquat (pq2) export, we analyzed fluxes from the StressME model under an optimized split ratio (i.e., 0.2 and 1.0 for the HPE and LPE strain, respectively) using the simulated full flux dataset (Dataset EV1) under different extracellular paraquat concentrations, where the growth rate (μ, h^-1^) and translation fluxes for EmrE (t_EmrE_ representing translation_b0543) and a pq2 reductase (t_Red_ representing translation_b3924) were extracted (mmol protein synthesized gDW^-1^ h^-1^).

pq2 fluxes were partitioned into import (v_import_), intracellular reduction (v_reduction_), and export (v_export_) through EmrE (all in mmol gDW^−1^ h^−1^). The energetic costs associated with transport were calculated as:

12 $${{\rm{PMF}}}\_{{\rm{Cost}}}=2\!\cdot \!{{{\rm{v}}}}_{{{\rm{export}}}}({{\rm{\mu }}}{{\rm{mol}}}\,{{{\rm{H}}}}^{+}\,{{{\rm{gDW}}}}^{-1}\,{{{\rm{h}}}}^{-1})$$

13 $${{\rm{ATP}}}\_{{\rm{Cost}}}={{{\rm{v}}}}_{{{\rm{import}}}}({{\rm{\mu }}}{{\rm{mol}}}\,{{\rm{ATP}}}\,{{{\rm{gDW}}}}^{-1}\,{{{\rm{h}}}}^{-1})$$

assuming two protons per pq2 exported and one ATP per pq2 imported. This framework enables direct assessment of the trade-off between energy and protein investment on pq2 export versus reduction via EmrE-mediated export and PQ2RED-mediated reduction, respectively.

### Data analysis, visualization, and statistics

Prism (GraphPad) version 10 was used for quantification and generation of plots. All experiments in this study were performed at least in triplicate. The figure legends of each plot mention the statistical details and tests used. Blinding was not performed for the experimental groups. Graphics for some figures were created using BioRender.com.

## Supplementary information

Appendix

Peer Review File

Dataset EV1

Source data Fig. 1

Source data Fig. 2

Source data Fig. 3

Source data Fig. 4

Source data Fig. 5

Source data Fig. 6

Appendix Figure S1 Source Data

## Data Availability

DNAseq and RNAseq data supporting this study are deposited in the NCBI SRA (PRJNA1404458) and GEO (GSE316857), respectively. The strains generated in this project are available for distribution for research purposes. The computational environment used for all simulations is available as a Docker container (stressme v1.2) from Docker Hub at https://hub.docker.com/r/queensysbio/stressme/tags (Tag: v1.2; Digest: sha256:b41b5348235dcd4cb37c608e89cca3e53d72ecb67bd29c5b3bf491f099e81d12). The flux solutions generated from the Low Paraquat Evolved (LPE) and High Paraquat Evolved (HPE) simulations are included in the Dataset EV1. The source data of this paper are collected in the following database record: biostudies:S-SCDT-10_1038-S44319-026-00933-y.
