# Supplementary material for: Selection favors cost-ordered adaptive mutational paths in a stress-magnitude-dependent manner
Source: EMBO Rep. 2026 Sep 12;27(20):6050–60. doi: 10.1038/s44319-026-00933-y (PMC13694702; doi:10.1038/s44319-026-00933-y)

This file includes:

Appendix Tables
Appendix Table S1 : Sample code used during running the ALE experiment (Page no. 1)
Appendix Table S2 : List of genes mutated in the paraquat evolved strains (Page no. 2)
Appendix Table S3 : Summary of the modifications introduced in StressME v1.2 relative to StressME v1.1. (Page no. 3)
Appendix Table S4 : Summary of the role of each model quantity, distinguishing calibrated parameters, experimental inputs, intermediate calculations, and ME model predictions. (Page no. 3)

Appendix Figures
Appendix Figure S1 : Impact of paraquat on the growth of GMOS, LPE and HPE strains. (Page no. 4)

Appendix Table S1 : Sample code used during running the ALE experiment

**Strain**	**ALE code**
LPE-A	*E. coli*_A9F60I1
LPE-B	*E. coli*_A10F60I1
LPE-C	*E. coli*_A12F60I1
HPE-A	*E. coli*_A25F45I1
HPE-B	*E. coli*_A26F45I1
HPE-C	*E. coli*_A27F45I1
LH1PE-A	*E. coli*_A29F40I1
LH1PE-B	*E. coli*_A30F40I1
LH1PE-C	*E. coli*_A31F40I1
LH2PE-A	*E. coli*_A37F30I1
LH2PE-B	*E. coli*_A38F30I1
LH2PE-C	*E. coli*_A39F30I1

Appendix Table S2 : List of genes mutated in the paraquat evolved strains

Strain	Mutations compared to WT *E. coli* strain			
LPE-A	*rpoB*<br>Y555H	*potC*<br>IS2 Coding (497-501/795 nt)		
LPE-B	*rpoB*<br>H526Y	*potD*<br>Δ1bp Coding (260/1047 nt)		
LPE-C	Δ82bp<br>*pyrE*→/→*rph*	*potA*<br>G55D		
HPE-A	*rpoB*<br>S712C		*renD*→/→*emrE*<br>IS1 Intergenic<br>(+21/-40)	
HPE-B	*rpoB*<br>S712C		*renD*→/→*emrE*<br>IS1 Intergenic<br>(+21/-40)	
HPE-C	*rpoB*<br>H526Y		*renD*→/→*emrE*<br>IS1 Intergenic<br>(+21/-39)	
LH1PE-A	*rpoB*<br>H526Y	*potD*<br>Δ1bp Coding (260/1047 nt)	*renD*→/→*emrE*<br>IS1 Intergenic<br>(+21/-39)	
LH1PE-B	*rpoB*<br>H526Y	*potD*<br>Δ1bp Coding (260/1047 nt)	*renD*→/→*emrE*<br>IS1 Intergenic<br>(+21/-39)	
LH1PE-C	*rpoB*<br>H526Y	*potD*<br>Δ1bp Coding (260/1047 nt)	*renD*→/→*emrE*<br>IS1 Intergenic<br>(+21/-39)	
LH2PE-A	*rpoB*<br>H526Y	*potD*<br>Δ1bp Coding (260/1047 nt)	*renD*→/→*emrE*<br>IS1 Intergenic<br>(+21/-39)	*ygfZ*<br>A65V
LH2PE-B	*rpoB*<br>H526Y	*potD*<br>Δ1bp Coding (260/1047 nt)	*renD*→/→*emrE*<br>IS1 Intergenic<br>(+21/-39)	*sdhE*←/→*ygfZ*<br>Intergenic (-232/-11)
LH2PE-C	*rpoB*<br>H526Y	*potD*<br>Δ1bp Coding (260/1047 nt)	*renD*→/→*emrE*<br>IS1 Intergenic<br>(+21/-39)	*ygfZ*<br>N215D

Appendix Table S3 : Summary of the modifications introduced in StressME v1.2 relative to StressME v1.1.

Category	StressME v1.1	StressME v1.2
Paraquat metabolites	Not included	pq2_e, pq2_p, pq2_c
Paraquat transport reactions	Not included	PotABCD import, EmrE export, exchange and transport reactions
Protein complexes	Original StressME complexes	Added PotABCD paraquat transporter and EmrE transporter complexes
Expression constraints	Original StressME genes	Added expression coupling for paraquat reductase (b3924) and EmrE (b0543)
Uptake kinetics	Not included	Calibrated Michaelis–Menten uptake (V_{max}, K_m)
Paraquat cycling	Not included	Parameterized $v_{reduction} = f_{red} v_{import}$
ROS input	Native StressME ROS	Paraquat-induced superoxide burden parameterized from $v_{reduction}$
Transport energetics	Native transport costs	Added ATP cost of PotABCD import and PMF cost of EmrE export
Oxidative-stress response	Original StressME	Original StressME driven by paraquat-specific ROS input

Appendix Table S4 : Summary of the role of each model quantity, distinguishing calibrated parameters, experimental inputs, intermediate calculations, and ME model predictions.

Quantity	Role
Vmax	calibrated parameter
Km	calibrated parameter
fred	calibrated parameter
External PQ	experimental input
v_{import}	computed from Michaelis–Menten kinetics
$v_{reduction}$	computed from f_{red} x v_{import}
Superoxide burden	parameterized from $v_{reduction}$
ATP cost	predicted by StressME
PMF cost	predicted by StressME
Detoxification proteins	predicted by StressME
Repair proteins	predicted by StressME
Growth rate	predicted by StressME

Appendix Figure S1 : Impact of paraquat on the growth of GMOS, LPE and HPE strains. The growth curve shows a mean of three biological replicates (with three technical replicates each), and the error bars show the standard error of the mean.

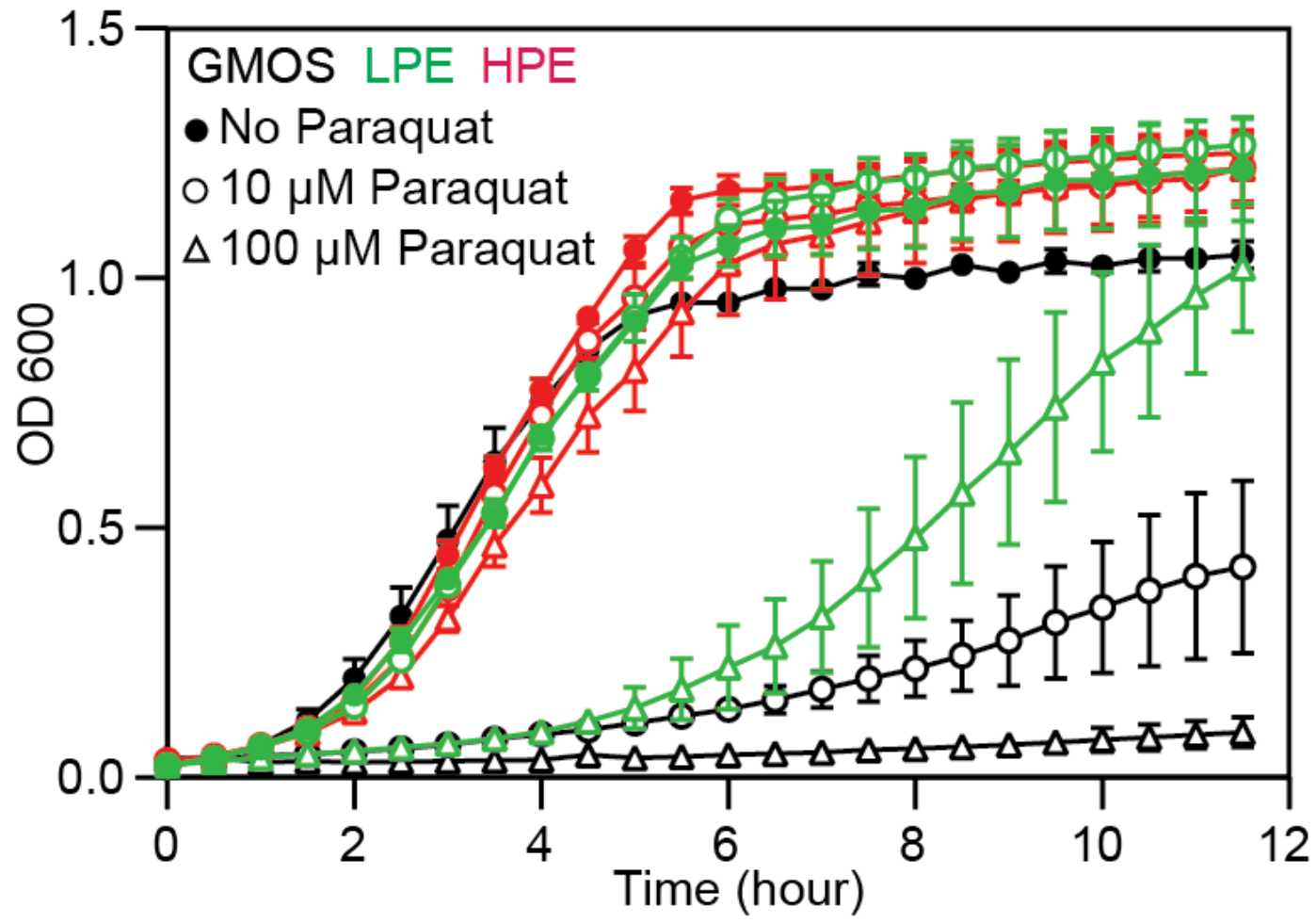

Supplement: Supplementary file 1 [file 44319_2026_933_MOESM1_ESM.pdf]
