# Supplementary material for: Selection favors cost-ordered adaptive mutational paths in a stress-magnitude-dependent manner
Source: EMBO Rep. 2026 Sep 12;27(20):6050–60. doi: 10.1038/s44319-026-00933-y (PMC13694702; doi:10.1038/s44319-026-00933-y)
Supplement: Supplementary file 2 [file 44319_2026_933_MOESM2_ESM.pdf]

EMBO reports

Selection favors cost-ordered adaptive mutational paths in a stress-magnitude-dependent manner

Anjali Patil, Jiao Zhao, Snehal Khairnar, Sonali Srivastava, Laurence Yang, and Amitesh Anand

Corresponding author(s): Amitesh Anand (amitesh.anand@tifr.res.in)

Review Timeline:

Submission Date:	17th Mar 26
Editorial Decision:	2nd Apr 26
Appeal Received:	13th Apr 26
Editorial Decision:	1st Jun 26
Revision Received:	7th Jul 26
Editorial Decision:	18th Aug 26
Revision Received:	21st Aug 26
Accepted:	31st Aug 26

Editor: Kurt Weir / Esther Schnapp

Transaction Report:

(Note: With the exception of the correction of typographical or spelling errors that could be a source of ambiguity, letters and reports are not edited. Depending on transfer agreements, referee reports obtained elsewhere may or may not be included in this compilation. Referee reports are anonymous unless the Referee chooses to sign their reports.)

2nd Apr 2026
Dear Dr. Anand,

Thank you for the submission of your manuscript to EMBO reports. I apologize for my delayed response, but I have now carefully assessed your paper. In addition, I have also consulted with an advisor with expertise in directed evolution in the laboratory. I am sorry to say that our conclusion from these considerations was that we cannot offer publication in EMBO reports.

We appreciate your finding that E. choli display distinct sets of adaptive mutations at different concentrations of paraquat and that this is explained by the metabolic cost of different adaptations.

As indicated above, we have sought expert advice on your manuscript. The advisor we contacted has meanwhile replied and found that the findings you report here, though well demonstrated, are similar to what has already been reported in the field. Please see below my signature for a quote.

Taking the concerns of the advisor into account, we have decided to not continue with in-depth peer review.

Although we are not able to consider your manuscript for publication in EMBO Reports, we think it is an excellent candidate for our not-for-profit open access sister journal, Life Science Alliance (http://www.life-science-alliance.org/; a broad scope journal published in partnership between the EMBO-, Rockefeller University-, and Cold Spring Harbor Laboratory Presses).

Should you transfer, LSA editors will send your manuscript out for peer-review. All files and manuscript details will be automatically transferred, and you will have the opportunity to include specific comments or requests for the LSA editors before finalizing transfer.

Tim Fessenden, Executive Editor of Life Science Alliance (t.fessenden@life-science-alliance.org), will be pleased to answer any questions.

Yours sincerely,

Kurt Weir
Editor
EMBO Reports

• "There have been other studies, mostly looking at how microbes gain antibiotic resistance, that show exactly what is reported here - mild stress leads to different adaptive paths that have lower fitness costs compared to the mutations that are required to become resistant to higher concentrations of antibiotics."

** As a service to authors, EMBO Press provides authors with the ability to transfer a manuscript that one journal cannot offer to publish to another journal, without the author having to upload the manuscript data again. To transfer your manuscript to another EMBO Press journal using this service, please click on Link Not Available

Dear Kurt,

Thank you for your email.

While I have to accept the decision, it is disappointing that despite taking such a long time the feedback is relatively superficial. There are two strengths of the manuscript (1) identification of flux-regulation based oxidative stress mechanisms and (2) explanation of non-overlapping adaptive strategies based on metabolic cost.

I am ofcourse fully aware of the works on antibiotic resistance that is being refered here. To the best of my knowledge, none of those studies have provided any metabolic justification or explanation and ofcoure no cost estimation. Infact I had a close dialogue with one of the leading researchers in this direction and they themselves were excited to see the energy budgting aspect of our study. Here is the concluding remark from their work:

"We find that evolution of gene dosage depends on expression demand, which is generated by antibiotic and exacerbated by proteolysis of drug-resistant mutants of DHFR. We propose a novel role for proteostasis as a determinant of copy number evolution in antibiotic-resistant bacteria."

And the mechanistic aspect of our work has been overlooked.

I am not sure if this explanation will make you revert your decision, but I sincerely wanted to highlight the gap in current editorial review. I understand your challenges of dealing with large volume of submissions, but this kind of response demotivates the young scientists who have worked hard to produce this story.

Best,

Amitesh

Dear Dr. Anand

Thank you for the submission of your research manuscript to our journal. We have now received the full set of referee reports that is copied below.

As you will see, the referees acknowledge that the findings are interesting and that the conclusions are overall supported by the data presented but they also raise a number of concerns and have suggestions how to further strengthen the data, particularly more carefully framing your conclusions in the context of prior literature and your results.

Given these constructive comments, we would like to invite you to revise your manuscript with the understanding that the referee concerns (as detailed above and in their reports) must be fully addressed and their suggestions taken on board. Please address all referee concerns in a complete point-by-point response. Acceptance of the manuscript will depend on a positive outcome of a second round of review. It is EMBO Reports policy to allow a single round of revision only and acceptance or rejection of the manuscript will therefore depend on the completeness of your responses included in the next, final version of the manuscript.

We realize that it is difficult to revise to a specific deadline. In the interest of protecting the conceptual advance provided by the work, we recommend a revision within 3 months (September 1st). Please discuss the revision progress ahead of this time with the editor if you require more time to complete the revisions.

I am also happy to discuss the revision further via e-mail or a video call, if you wish.

You can either publish the study as a short report or as a full article. For short reports, the revised manuscript should not exceed 27,000 characters (including spaces but excluding materials & methods and references) and 5 main plus 5 expanded view figures. The results and discussion sections must further be combined, which will help to shorten the manuscript text by eliminating some redundancy that is inevitable when discussing the same experiments twice. For a normal article there are no length limitations, but it should have more than 5 main figures and the results and discussion sections must be separate. In both cases, the entire materials and methods must be included in the main manuscript file.

================
IMPORTANT NOTE:

We perform an initial quality control of all revised manuscripts before re-review. Your manuscript will FAIL this control and the handling will be delayed IN CASE the following APPLIES:

1) A data availability section providing access to data deposited in public databases is missing. If you have not deposited any data, please add a sentence to the data availability section that explains that.

2) Your manuscript contains statistics and error bars based on n=2. Please use scatter blots in these cases. No statistics should be calculated if n=2.

When submitting your revised manuscript, please carefully review the instructions that follow below. Failure to include requested items will delay the evaluation of your revision.

================

When submitting your revised manuscript, we will require:

1) a .docx formatted version of the manuscript text (including legends for main figures, EV figures and tables). Please make sure that the changes are highlighted to be clearly visible.

2) individual production quality figure files as .eps, .tif, .jpg (one file per figure).
Please download our Figure Preparation Guidelines (figure preparation pdf) from our Author Guidelines pages https://www.embopress.org/page/journal/14693178/authorguide for more info on how to prepare your figures.

3) a .docx formatted letter INCLUDING the reviewers' reports and your detailed point-by-point responses to their comments. As part of the EMBO Press transparent editorial process, the point-by-point response is part of the Review Process File (RPF), which will be published alongside your paper.

4) a complete author checklist, which you can download from our author guidelines (<https://www.embopress.org/page/journal/14693178/authorguide>). Please insert information in the checklist that is also

reflected in the manuscript. The completed author checklist will also be part of the RPF.

5) Please note that all corresponding authors are required to supply an ORCID ID for their name upon submission of a revised manuscript (<https://orcid.org/>). Please find instructions on how to link your ORCID ID to your account in our manuscript tracking system in our Author guidelines (<https://www.embopress.org/page/journal/14693178/authorguide#authorshipguidelines>)

6) We replaced Supplementary Information with Expanded View (EV) Figures and Tables that are collapsible/expandable online. A maximum of 5 EV Figures can be typeset. EV Figures should be cited as 'Figure EV1, Figure EV2" etc... in the text and their respective legends should be included in the main text after the legends of regular figures.

- For the figures that you do NOT wish to display as Expanded View figures, they should be bundled together with their legends in a single PDF file called *Appendix*, which should start with a short Table of Content. Appendix figures should be referred to in the main text as: "Appendix Figure S1, Appendix Figure S2" etc. See detailed instructions regarding expanded view here: <https://www.embopress.org/page/journal/14693178/authorguide#expandedview>

- Additional Tables/Datasets should be labeled and referred to as Table EV1, Dataset EV1, etc. Legends have to be provided in a separate tab in case of .xls files. Alternatively, the legend can be supplied as a separate text file (README) and zipped together with the Table/Dataset file.

7) Before submitting your revision, primary datasets (and computer code, where appropriate) produced in this study need to be deposited in an appropriate public database (see < https://www.embopress.org/page/journal/14693178/authorguide#dataavailability>).

Please remember to provide a reviewer password if the datasets are not yet public.

The accession numbers and database should be listed in a formal "Data Availability " section (placed after Materials & Method) that follows the model below (see also < https://www.embopress.org/page/journal/14693178/authorguide#dataavailability>). Please note that the Data Availability Section is restricted to new primary data that are part of this study.

Data availability

The datasets (and computer code) produced in this study are available in the following databases:

- RNA-Seq data: Gene Expression Omnibus GSE46843 (https://www.ncbi.nlm.nih.gov/geo/query/acc.cgi?acc=GSE46843)
- [data type]: [name of the resource] [accession number/identifier/doi] ([URL or identifiers.org/DATABASE:ACCESSION])

*** Note - All links should resolve to a page where the data can be accessed. ***

8) At EMBO Press we ask authors to provide source data for the main manuscript figures. You will receive a separate email with instructions for providing source data with your revised manuscript, including how to upload and organize the files.

Additional information on source data and instruction on how to label the files are available <https://www.embopress.org/page/journal/14693178/authorguide#sourcedata>

9) The journal requires a statement specifying whether or not authors have competing interests (defined as all potential or actual interests that could be perceived to influence the presentation or interpretation of an article). In case of competing interests, this must be specified in your disclosure statement. Further information: https://www.embopress.org/competing-interests

10) Figure legends and data quantification:
The following points must be specified in each figure legend:

- the name of the statistical test used to generate error bars and P values,
- the EXACT p-values,
- the number (n) of independent experiments (please specify technical or biological replicates) underlying each data point,
- the nature of the bars and error bars (s.d., s.e.m.)

- If the data are obtained from n {less than or equal to} 5, show the individual data points in addition to the SD or SEM.
- If the data are obtained from n {less than or equal to} 2, use scatter blots showing the individual data points.

Discussion of statistical methodology can be reported in the materials and methods section, but figure legends should contain a basic description of n, P and the test applied.

See also the guidelines for figure legend preparation:

https://www.embopress.org/page/journal/14693178/authorguide#figureformat

- Please also include scale bars in all microscopy images and define their size in the legend, not the image.

11) Our journal encourages inclusion of *data citations in the reference list* to directly cite datasets that were re-used and obtained from public databases. Data citations in the article text are distinct from normal bibliographical citations and should directly link to the database records from which the data can be accessed. In the main text, data citations are formatted as follows: "Data ref: Smith et al, 2001" or "Data ref: NCBI Sequence Read Archive PRJNA342805, 2017". In the Reference list, data citations must be labeled with "[DATASET]". A data reference must provide the database name, accession number/identifiers and a resolvable link to the landing page from which the data can be accessed at the end of the reference. Further instructions are available at <https://www.embopress.org/page/journal/14693178/authorguide#referencesformat>.

12) All Materials and Methods need to be described in the main text using our 'Structured Methods' format. According to this format, the Methods section includes a Reagents and Tools Table (listing key reagents, experimental models, software and relevant equipment and including their sources and relevant identifiers) followed by a Methods and Protocols section describing the methods, ideally using a step-by-step protocol format. The aim is to facilitate adoption of the methodologies across labs. Please download and fill our Reagents and Tools Table template (.docx), which you can find in our author guidelines: https://www.embopress.org/page/journal/14693178/authorguide#structuredmethods.
When submitting your revised manuscript, please do not include the Reagents and Tools Table in the Methods section of the manuscript but upload it as a separate file choosing the file type "Reagent Table".
An example of a Method paper with Structured Methods can be found here: https://www.embopress.org/doi/10.15252/msb.20178071.

13) As part of the EMBO publication's Transparent Editorial Process, EMBO Reports publishes online a Review Process File to accompany accepted manuscripts. This File will be published in conjunction with your paper and will include the referee reports, your point-by-point response and all pertinent correspondence relating to the manuscript.

You are able to opt out of this by letting the editorial office know (emboreports@embo.org). If you do opt out, the Review Process File link will point to the following statement: "No Review Process File is available with this article, as the authors have chosen not to make the review process public in this case."

We would also welcome the submission of cover suggestions, or motifs to be used by our Graphics Illustrator in designing a cover.

I look forward to seeing a revised form of your manuscript when it is ready and please let me know if you have questions or comments regarding the revision.

Yours sincerely,

Kurt Weir
Editor
EMBO Reports

Referee #1:

Summary

The authors evolve E. coli K-12 MG1655 at 10 µM and 100 µM paraquat in triplicate, re-evolve the low-paraquat background at 100 µM and 200 µM, and recover three mutation classes: loss-of-function lesions in the potD-potABC polyamine ABC importer at 10 µM, an IS1 insertion upstream of emrE at 100 µM, and alleles near ygfZ at 200 µM. Targeted deletions of potD, potF, and emrE support the directional interpretation, a StressME extension computes smaller transport cost for the low-paraquat strategy, and a competition assay with WTΔpotD as a surrogate supports a fitness advantage at 10 µM.

The dose-stratified trajectory is a useful contribution and the core phenotypic dataset is sound. I recommend minor revision, focused on four areas of framing and interpretation: more direct engagement with the authors' own earlier paraquat ALE (Rychel et al., 2023) and the older paraquat-polyamine biochemistry, two additional knockout controls, reframing Figure 5 as parameterized cost accounting, and reconciling two ygfZ references that report different phenotypes. Each concern ends with two possible paths, one experimental and one interpretive, so the revision can proceed either way.

Major concerns

1. Explicit attribution to the authors' earlier paraquat-ALE findings would strengthen the Results framing

The connection to older paraquat-polyamine biochemistry is worth making more direct. Kashiwagi et al. (1990), currently cited as part of a bundled [17-19] citation alongside Qiao et al. (2024, structural) and Fujita et al. (2012, Arabidopsis LAT family), provides the most direct biochemical evidence for the claim that the polyamine transporter is "hijacked for paraquat influx": their Table II shows that 50 µM paraquat inhibits pPT104-mediated putrescine uptake by 99.5 percent on the same potABCD operon recovered by LOF lesions in this ALE. Two additional primary references that would strengthen this passage are Kao and Hassan (1985), who measured active [^{14}C]paraquat uptake and characterized a paraquat-tolerant mutant with altered uptake kinetics, and Minton, Tabor, and Tabor (1990), who showed that paraquat toxicity is increased more than 10-fold in E. coli strains defective in spermidine biosynthesis and proposed competition between spermidine and paraquat for intracellular polyanionic binding sites. Neither is in the current bibliography. Fujita et al. (2012) is an Arabidopsis LAT/APC superfamily paper whose structural analogy is to AdiC, a bacterial amino-acid antiporter; LAT/APC and potABCD/ABC are different transporter superfamilies, so it is not the most appropriate structural anchor for a bacterial ABC importer.

The emrE finding directly parallels a result in the authors' earlier paraquat ALE. Rychel et al. (2023), which shares senior authorship (Anand, Yang) with the present manuscript, reported that two of three first-generation paraquat-evolved lineages (1_0 and 2_0) acquired approximately 42-fold amplification in the emrE region (their Figure 3A), and that the third lineage (3_0) and its descendants acquired a 9 bp insertion 39 bp upstream of emrE consistent with an IS1-mediated event. The Patil HPE IS1 insertion at +21/-39 in the renD-emrE intergenic region appears to be a convergent recovery of the 3_0-class mechanism. Rychel et al. (2023) is already cited at this point in the Results (the [10,23,24] cluster) for the general mechanism that IS insertions can alter downstream gene expression, but the specific emrE-activation precedent is not discussed. A sentence that directly ties the HPE IS1 event to the emrE-activation observation in the authors' earlier paper would make the convergence visible to readers.

Either (a) directly measure intracellular paraquat in WT, WTΔpotD, LPE, HPE, and HPE ΔemrE to test whether entry is reduced and efflux is elevated as predicted, or (b) narrow the Results section 2 claims to describe the LPE outcome as an ALE recovery of the previously characterized paraquat-polyamine uptake interaction (Kao & Hassan, 1985; Kashiwagi et al., 1990; Minton et al., 1990), and present the HPE IS1 insertion as a convergent recovery of the IS1-mediated emrE-activation mechanism first observed in Rychel et al. (2023), lineage 3_0.

2. Two targeted-knockout controls are missing

WTΔemrE baseline paraquat sensitivity is not tested. The ΔemrE experiment is done only in the evolved HPE background (Figure 3C). The manuscript's own data show that the IS1 insertion elevates emrE transcript in HPE relative to WT (Figure 3B). emrE was originally identified as a methyl-viologen antiporter through gain-of-function experiments in which overexpression confers paraquat resistance (Yerushalmi et al., 1995; Morimyo et al., 1992). Two interpretations of the Figure 3C result are therefore compatible with the data: loss of the IS1-driven overexpression state, or loss of emrE function more generally. Adding a WTΔemrE growth curve at 100 µM paraquat alongside the HPE and HPEΔemrE curves would distinguish these directly.

Polyamine pool in ΔpotD is not measured. Minton et al. (1990) established that spermidine specifically (not putrescine) protects E. coli against paraquat toxicity: 10 µM spermidine abolished paraquat growth inhibition in polyamine-deficient strains, whereas putrescine required 1500 µM, and they proposed competition between spermidine and paraquat for polyanionic binding sites (DNA, ribosomes) rather than ROS scavenging. ΔpotD in this work may therefore affect paraquat tolerance through reduced paraquat entry, through altered intracellular spermidine, or through both, and these three scenarios are not equivalent.

Either add WTΔemrE growth curves at 0, 10, and 100 µM paraquat and measure intracellular spermidine and putrescine in WT, ΔpotD, and ΔpotDΔpotF, or note these uncontrolled variables explicitly in the Discussion and soften the mechanistic attribution for both the potD and the emrE lines.

3. The StressME cost analysis would benefit from additional parameterization

The StressME extension is useful as parameterized cost accounting. As a genome-scale prediction it has four issues; reframing Figure 5 as parameterized cost accounting would resolve them.

3a. frac_red does not enter the cost calculation. Equation 6 imposes v_import = v_reduction + v_export – v_oxidation under the steady-state assumption v_reduction = v_oxidation. This gives v_export = v_import identically, so PMF cost = 2·v_import and ATP cost = v_import regardless of frac_red. The optimized values of 0.2 (HPE) and 1.0 (LPE) cannot affect the cost bars in Figure 5B. The split-ratio description in Methods should be corrected, or the model reformulated so PQ1OX carries flux and ROS burden varies with frac_red.

3b. Vmax and Km are not identifiable from two dose points. Two paraquat concentrations (10 µM and 100 µM) cannot separately constrain a two-parameter Michaelis-Menten model. When [PQ] is below Km, v_import is approximately (Vmax/Km)·[PQ] and only the ratio is identified. Assigning LPE a higher Km than HPE is therefore equivalent to assigning it lower cost in the tested

range. The cost ranking in Figure 5B follows from the chosen parameter pair rather than from genome-scale inference.

3c. Absolute cost magnitude is not benchmarked. The transport costs in Figure 5B are small relative to ATP maintenance flux in a standard E. coli ME solution. Connecting the six-fold relative cost difference to the competition outcome in Figure 2D would be stronger with a magnitude check: fitting the selection coefficient s from the competition time-course and converting the StressME transport-cost difference into an equivalent growth-rate penalty via the ME proteome-cost model would let the two be compared directly.

3d. PQ1OX carries zero flux in the flux solution. The diagram in Figure 5A depicts a simulated redox-cycling pathway, but the solver does not carry oxidation flux; superoxide burden is imposed through an external bound rather than generated by the redox cycle. Either Figure 5A should be presented as a cost-accounting schematic, or the model should be run with PQ1OX active and superoxide coupled to v_oxidation.

Either (a) reparameterize with direct [C14]paraquat uptake kinetics across at least five concentrations, activate PQ1OX, and report sensitivity of the cost ranking to Vmax, Km, frac_red, and emrE stoichiometry, or (b) present Figure 5 as a parameterized cost estimate rather than a genome-scale prediction, state which fluxes are fixed inputs versus solver outputs, and temper the Abstract and Conclusion accordingly. Code and data availability are very well handled (StressME v1.2 Docker container and LPE/HPE flux solutions in Dataset S1). A brief supplementary methods note describing what changed from v1.1 to v1.2, and flux solutions for all four lineage-dose combinations, would complete the picture.

4. The ygfZ interpretation would benefit from reconciling two cited references that report different phenotypes

4a. The Lin et al. (2010) paraquat result differs from how reference 32 is used. Lin et al. tested paraquat explicitly and report that "when paraquat was applied at 1.28 μg per disc, the ΔygfZ strain showed the same resistance as the parental strain whereas the inhibition zone of the ΔsodA strain increased substantially." They further show that ygfZ overexpression does not suppress paraquat-induced superoxide (their Figure 3C), and their Acknowledgements section states "this is an independent study and has no connection to the recent report by Waller et al." The manuscript cites reference 32 for "ygfZ has been implicated in oxidative stress mitigation," which does not fit the paraquat finding in Lin et al.

4b. Waller et al. (2010) does not experimentally characterize IBA57. The IBA57 claim in Waller et al. is inherited from Gelling et al. (2008). Waller et al. also explicitly favor a "repair of specific enzymes as opposed to de novo synthesis of all Fe/S clusters" framing, so the manuscript's statement that "IBA57 is involved in the assembly of the Fe-S cluster" would be more accurate citing Gelling et al. (2008) directly and saying "repair" rather than "assembly." Mühlenhoff et al. (2022) further report that IBA57 function in mitochondrial [4Fe-4S] maturation is tetrahydrofolate-independent and tentatively extend that conclusion to bacterial ygfZ.

4c. Two cited references contradict each other on the ΔygfZ-paraquat phenotype. Waller et al. (2010) report the deletant "was more sensitive to both stress agents" (their Fig. S3A); Lin et al. (2010) report identical paraquat resistance. This contradiction is not addressed. It also matters for the mechanistic story: an LOF allele producing paraquat tolerance is hard to reconcile with the Waller sensitivity phenotype.

4d. The LH2PE-B intergenic allele (-232/-11) sits in a divergent promoter shared with sdhE. sdhE (b2897) and ygfZ (b2898) are head-to-head, separated by 243 bp. Supp Table 2 annotates this variant as "sdhE←/→ygfZ Intergenic (-232/-11)," acknowledging the divergent-promoter context, but the main-text interpretation groups it with the ygfZ coding mutations. RNA-seq data are deposited (GEO GSE316857), so reporting sdhE and ygfZ transcript ratios in LH2PE-B relative to LH1PE would distinguish whether the intergenic allele acts via sdhE, ygfZ, or both. Rychel et al. (2023) identify the sdhABCD Fe-S clusters as targets of ROS damage in paraquat-evolved strains (their Figure 6F), so sdhE is a plausible target.

Either reconstruct A65V and N215D in WT MG1655 and in LH1PE to test chassis dependence, measure sdhE and ygfZ transcripts in LH2PE-B, or state that LH2PE recovered mutations at the sdhE-ygfZ locus consistent with prior paraquat ALE studies (Rychel et al., 2023; Phaneuf et al., 2024, which compiled 46 ygfZ mutations from ALEdb with L29R, V107E, and T108P as the paraquat-frequent subset clustering near the ethandiol binding sites) and that the specific mechanistic contribution of each allele remains to be determined.

Technical and editorial points

1. Statistical reporting on emrE expression. The Kruskal-Wallis $P = 0.0253$ with $n = 3$ is fragile, and the transcript ratio is carried forward into Equation 8 as the basis for the emrE catalytic-rate inference. Reporting $\log_2$ fold change with 95 percent confidence intervals for each lineage and adding RT-qPCR validation would strengthen this.

2. Competition assay uses an allele-level proxy. WTΔpotD is used as the LPE surrogate and HPE-A as competitor, but WTΔpotD does not carry the growth-promoting rpoB and pyrE-rph mutations that LPE strains acquired. Either repeating with bona fide LPE and HPE lineages or stating this as an allele-level proxy would be appropriate.

3. IS1 insertion versus amplification in HPE. Given the Rychel et al. (2023) precedent of 42× amplification flanking IS3 elements in two of three lineages, verifying by qPCR or read-depth analysis that the HPE IS1 event is a single-copy insertion rather than a breakpoint of a tandem amplification would rule out the amplification alternative.

4. The abstract would read more accurately if it acknowledged that the potD-potABC paraquat-uptake connection has a long-standing biochemical literature (Kao & Hassan, 1985; Kashiwagi et al., 1990; Minton et al., 1990) and that IS-mediated emrE activation under paraquat ALE was previously observed by Rychel et al. (2023). Framing the StressME output as a parameterized cost calculation, rather than an emergent genome-scale prediction, would also match what Figure 5 actually delivers.

Summary recommendation

The phenotypic dataset is sound and the dose-stratified trajectory is a useful contribution. Four areas of framing and interpretation would benefit from revision: (i) explicit attribution to the authors' earlier paraquat ALE (Rychel et al., 2023) and to the older paraquat-polyamine biochemistry (Kao & Hassan, 1985; Kashiwagi et al., 1990; Minton et al., 1990); (ii) two additional knockout controls (WTΔemrE and polyamine-pool measurements in ΔpotD); (iii) addressing the four StressME issues in Figure 5 and reframing it as parameterized cost accounting; and (iv) reconciling the Lin et al. versus Waller et al. discrepancy and separating the sdhE-ygfZ intergenic allele from the ygfZ coding mutations.

References

Fujita, M., Fujita, Y., Iuchi, S., Yamada, K., Kobayashi, Y., Urano, K., Kobayashi, M., Yamaguchi-Shinozaki, K., & Shinozaki, K. (2012). Natural variation in a polyamine transporter determines paraquat tolerance in Arabidopsis. Proceedings of the National Academy of Sciences, 109(16), 6343-6347.
Gelling, C., Dawes, I. W., Richhardt, N., Lill, R., & Mühlenhoff, U. (2008). Mitochondrial Iba57p is required for Fe/S cluster formation on aconitase and activation of radical SAM enzymes. Molecular and Cellular Biology, 28(5), 1851-1861.
Kao, S. M., & Hassan, H. M. (1985). Biochemical characterization of a paraquat-tolerant mutant of Escherichia coli. Journal of Biological Chemistry, 260(19), 10478-10481.
Kashiwagi, K., Hosokawa, N., Furuchi, T., Kobayashi, H., Sasakawa, C., Yoshikawa, M., & Igarashi, K. (1990). Isolation of polyamine transport-deficient mutants of Escherichia coli and cloning of the genes for polyamine transport proteins. Journal of Biological Chemistry, 265(34), 20893-20897.
Lin, C.-N., Syu, W.-J., Sun, W.-S. W., Chen, J.-W., Chen, T.-H., Don, M.-J., & Wang, S.-H. (2010). A role of ygfZ in the Escherichia coli response to plumbagin challenge. Journal of Biomedical Science, 17, 84.
Minton, K. W., Tabor, H., & Tabor, C. W. (1990). Paraquat toxicity is increased in Escherichia coli defective in the synthesis of polyamines. Proceedings of the National Academy of Sciences, 87(7), 2851-2855.
Morimyo, M., Hongo, E., Hama-Inaba, H., & Machida, I. (1992). Cloning and characterization of the mvrC gene of Escherichia coli K-12 which confers resistance against methyl viologen toxicity. Nucleic Acids Research, 20(12), 3159-3165.
Mühlenhoff, U., Dreher, L., Gräßler, B., Schulz, D., Altegoer, F., Schindelin, H., Bange, G., & Lill, R. (2022). The iron-sulfur cluster assembly ISC protein Iba57 executes a tetrahydrofolate-independent function in the maturation of [4Fe-4S] proteins. Journal of Biological Chemistry, 298(10), 102465.
Phaneuf, P. V., Lloyd, C. J., Catoiu, E., Lewis, N. E., Sandberg, T. E., Yang, L., Feist, A. M., & Palsson, B. O. (2024). Meta-analysis-driven strain design for mitigating oxidative stresses important in biomanufacturing. ACS Synthetic Biology, 13(7), 2045-2059.
Qiao, X., Wang, Q., Wang, X., Li, J., & Shi, Y. (2024). Structural insights into polyamine spermidine uptake by the ABC transporter PotABCD. Science Advances, 10(38), eado8107.
Rychel, K., Tan, J., Patel, A., Lamoureux, C., Hefner, Y., Szubin, R., Johnsen, J., Mohamed, E. T. T., Phaneuf, P. V., Anand, A., Olson, C. A., Park, J. H., Sastry, A. V., Yang, L., Feist, A. M., & Palsson, B. O. (2023). Laboratory evolution, transcriptomics, and modeling reveal mechanisms of paraquat tolerance. Cell Reports, 42(9), 113105.
Waller, J. C., Alvarez, S., Naponelli, V., Lara-Nuñez, A., Blaby, I. K., Da Silva, V., Ziemak, M. J., Vickers, T. J., Beverley, S. M., Edison, A. S., Rocca, J. R., Gregory, J. F. III, de Crécy-Lagard, V., & Hanson, A. D. (2010). A role for tetrahydrofolates in the metabolism of iron-sulfur clusters in all domains of life. Proceedings of the National Academy of Sciences, 107(23), 10412-10417.
Yerushalmi, H., Lebendiker, M., & Schuldiner, S. (1995). EmrE, an Escherichia coli 12-kDa multidrug transporter, exchanges toxic cations and H^+ and is soluble in organic solvents. Journal of Biological Chemistry, 270(12), 6856-6863.
Zhao, J., Patil, M., Khairnar, P. K., Srivastava, A., Yang, L., & Anand, A. (2024). StressME: unified computing framework of Escherichia coli metabolism, gene expression, and stress responses. PLoS Computational Biology.

Referee #2:

In this manuscript, Patil et al. have run Adaptive Laboratory Evolution (ALE) experiments with E. coli in the presence of two different concentrations of paraquat (10 µM = low dose; 100 µM = high dose), along with high- and low-dose challenges of WT, low paraquat-evolved (LPE) or high paraquat-evolved (HPE) sub-strains, and step-wise evolution. Whole-genome sequencing

was used to identify mutations underlying paraquat fitness and RNAseq was used to identify gene expression differences underlying paraquat fitness. Causality is verified for critical fitness genes by targeted mutagenesis. The outcome of this study is a very reasonable model in which adaption to low dose paraquat exposure occurs preferentially by mutation of the relatively low resource-demanding systems that transport paraquat into the cytosol whereas adaptation to high dose paraquat adaptation preferentially uses the higher resource-demanding activation of detoxification and damage-mediating systems. Interestingly, direct evolution in high dose does not appear to modify paraquat import but, along with the detox and damage-repair systems, it did upregulate a multidrug efflux pump that is thought to actively export paraquat.

Overall the experiments seem well designed and rigorous. The authors have made their sequence- and gene expression-data available on a public database; a statement of public availability of the evolved E. coli strains is missing.

Concerns.

1. The title, abstract, and much of the body of the paper state that this is a study of oxidative stress tolerance; however, oxidative stress is only indirectly studied. Instead, paraquat stress is studied. Paraquat treatment is an indirect inducer of oxidative stress; the outcomes of paraquat treatment are likely more complicated than simply inducing oxidative stress. This is largely overlooked in the submitted manuscript. Superoxide levels were not measured, only assumed or inferred. There are extended discussions and calculations in the Methods section in support of superoxide production in response to paraquat, but these remain theoretical, not empirical. There is a problem in the redox field and, indeed, extending to other fields that depend on the redox field for mechanistic understanding of redox processes, wherein oxidative stress serves, often without real evidence, as the "usual suspect", implicated for diverse impacts on biological systems wherein actual roles of oxidants are undemonstrated and tenuous. In human health, these implicated effects range from plausible, e.g., mutagenesis, to ridiculous, like aging. Whereas I don't dispute that superoxide radical anion is a byproduct of bacterial paraquat metabolism, it is neither the only product of this metabolism nor is its metabolism without other impacts on the cell. There is no doubt the authors know that it would be correct to have written this paper to precisely say that they are measuring "paraquat tolerance". They chose instead to phrase their title and conclusions on what they interpret from what they did and not what they did. I suspect this is done as a "sales-strategem", jumping on the bandwagon of naming oxidative stress as the usual suspect.

There are two possible resolutions: (1) re-write study study presenting all results and conclusions to precisely implicate paraquat resistance / toxicity and save the speculation about oxidative stress for its appropriate home, within a discrete Discussion section; or (2) rigorously quantitatively validate the roles of superoxide in this system, in the absence of other toxicities or physiological impacts, to fully rule out other influences, and then keep the text "oxidative stress"-focused. Option 2 might, however, be unachievable.

2. Other than one transcriptome heatmap (Fig. 4A), the only empirical measurements in the paper are growth rates. This results in many conclusions being tenuously supported by only indirect evidence. Examples:

a. Superoxide levels were not measured, only inferred. Measuring cellular superoxide and how it changes in each condition would strengthen the study.
b. Paraquat import, metabolism, and export is not measured, only inferred. Indeed, the roles of the PotD-PotABC polyamine-transporter as a paraquat transporter is inferred from the ability of a plant polyamine-transporter to transport paraquat but not directly tested or measured; it's role as the sole paraquat transporter is inferred from (non-impacted) growth characteristics of bacteria with disruption of PotF-PotGHI. Measuring cellular paraquat levels or paraquat metabolic flux in each condition would strengthen the study.
c. Whereas some of the mutations that were mapped would disrupt protein expression, some will make a protein but with a single amino acid change in the PotA ATP-binding domain is predicted to no longer transport paraquat. Transport, however, was not tested. Measuring paraquat transport rates of the WT PotD-PotABC and PotF-PotGHI, as well as the former with the PotA G55D mutation would strengthen the study.

3. I disagree with many of the authors' unnecessarily overly definitive statements, to the point that I find many cases wherein the results don't support the conclusions. As a few examples:

- p. 2, "We performed four separate adaptive laboratory evolutions (ALE) of Escherichia coli under different paraquat concentrations and with varied genetic backgrounds to delineate adaptive strategies to distinct magnitudes of superoxide stress." Problem: The introduction section awkwardly integrates general bacterial environment response with diverse aspects of pathogen-host interactions (not necessarily specific to bacterial pathogens nor are the bacterial responses necessarily exclusive of those in non-pathogenic bacteria). I think the above sentence needs to be preceded with a clarifying clause, such as: "To model potential responses of pathogenic bacteria to these oxidative stresses,..."
- p. 6, "... results ... indicate that PotF-PotGHI does not serve as the primary route for paraquat influx". Problem: PotF-PotGHI paraquat transport function was not measured. The results are consistent with...
- p. 8, "Our findings show that LPE partially decreases paraquat uptake, whereas HPE enhances its export alongside increased ROS detoxification." Problem: Neither decreased paraquat uptake, enhanced paraquat export, nor increased ROS detoxification were shown, but they are suggested by, or consistent with, the data.
- p. 10, "The simulation showed a difference in PMF and ATP requirements between LPE and HPE strains." Problem: A

simulation can allow predictions, not show differences. Neither PMF nor ATP were measured.

4. Minor:

a. p. 8, ETS is not defined.
b. p. 10, YgfZ is not defined / described
c. Fig. 5A seems to be missing an electron. NADPH is a 2-electron reactant but only a single radical product is shown.

Referee #3:

In this manuscript, Patil et al. used laboratory evolution against low (LPE) and high (HPE) concentrations of paraquat in E. coli and identified two unique mechanisms. For low concentrations of paraquat, LOF mutations in a polyamine transporter were recovered, suggesting that reduced influx is the mechanism of resistance. For high concentrations of paraquat, they observed an IS1 insertion upstream of emrE encoding at multidrug efflux pump, suggesting that paraquat export is necessary for resistance. The HPE mutants were resistant to low concentrations of paraquat, but were outcompeted by LPE cells in competition, suggesting a possible tradeoff. Through modeling, the authors propose that efflux pump upregulation has a higher cost in terms of both ATP costs and proton motive force, which makes sense for decreased import vs increased export. Finally, the authors sequentially evolved the LPE cells on 100 uM and then 200 uM paraquat, where they picked up the IS1 mutation and then another novel mutation in ygfZ that further improved resistance (though the mechanism is unclear).

I think this is an interesting paper, well written and with well-designed experiments. The logic is very clear. I just have one major philosophical disagreement with how the paper is framed. Throughout the paper, the authors frame the paper around "defense strategies" (sometimes hierarchical), and that E. coli "differentially engages modular stress-response programs depending on stress magnitude." This makes it sound like these are deliberate stress response programs, but they are not (unlike for example, the induction of pre-existing ROS defense genes that WT cells have already evolved for tolerating sub-lethal ROS). The cell does not evolve strategies that anticipate becoming a mutant in the future. I think a better framing that still includes the (interesting!) tradeoffs are the differences in evolutionary "paths" towards tolerance, and their predictability (very interesting that the authors obtained the same mutations across evolution experiments, especially the second ALE experiment with the LPE strains). Instead of "Cost adjusted hierarchical defense strategies enables stratified oxidative stress tolerance," something more like "Selection favors cost-ordered mutational paths in a stress-magnitude-dependent manner." I recommend that the authors go through the manuscript and re-frame where appropriate that this addresses which evolutionary paths are favored by selection, including not only tradeoffs but also mutational target size (likely affecting why the same mutations keep arising) and the repeatability of evolution (Stephen Jay Gould's "replaying the tape of life").

Minor critiques:

1) The authors use "flux" control, and it took me a minute to realize that they were talking about influx/efflux of paraquat and not metabolic flux. I think it would help to be more clear about that in places (including the abstract).

2) In the Methods, I think there should be a few more details about the lab evolution experiments. How many generations for each experiment? How were individual clones selected (just streaked on a plate at the end?), and do the supplemental tables represent every clone sequenced?

July 7, 2026

Manuscript ID: EMBOR-2026-64056V2-Q

Editor's/Referee's Comments: **Black Text**
Referee cross-comment: **Green Text**
Responses by authors: **Blue Text**

Editor's remark:
As you will see, the referees acknowledge that the findings are potentially interesting but they also raise a number of concerns and have suggestions on how to further strengthen the data, particularly more carefully framing your conclusions in the context of prior literature and your results.

We thank you for providing us with subject experts to review our manuscript. We are glad to note that all three referees consider the study interesting, rigorous, and publishable pending some modifications. We carefully considered all of the remarks and are presenting an appropriately revised manuscript.

Referee 1
Summary
The authors evolve E. coli K-12 MG1655 at 10 μM and 100 μM paraquat in triplicate, re-evolve the low-paraquat background at 100 μM and 200 μM, and recover three mutation classes: loss-of-function lesions in the potD-potABC polyamine ABC importer at 10 μM, an IS1 insertion upstream of emrE at 100 μM, and alleles near ygfZ at 200 μM. Targeted deletions of potD, potF, and emrE support the directional interpretation, a StressME extension computes a smaller transport cost for the low-paraquat strategy, and a competition assay with WTΔpotD as a surrogate supports a fitness advantage at 10 μM.

The dose-stratified trajectory is a useful contribution, and the core phenotypic dataset is sound. I recommend a minor revision, focused on four areas of framing and interpretation: more direct engagement with the authors' own earlier paraquat ALE (Rychel et al., 2023) and the older paraquat-polyamine biochemistry, two additional knockout controls, reframing Figure 5 as parameterized cost accounting, and reconciling two ygfZ references that report different phenotypes. Each concern ends with two possible paths, one experimental and one interpretive, so the revision can proceed either way.

Authors' response: Thank you for your encouraging remark. We have incorporated your suggestions into the revised manuscript.

Major concerns
1. Explicit attribution to the authors' earlier paraquat-ALE findings would strengthen the Results framing

The connection to older paraquat-polyamine biochemistry is worth making more direct. Kashiwagi et al. (1990), currently cited as part of a bundled [17-19] citation alongside Qiao et al. (2024, structural) and Fujita et al. (2012, Arabidopsis LAT family), provides the most direct biochemical evidence for the claim that the polyamine transporter is "hijacked for paraquat influx": their Table II shows that 50 µM paraquat inhibits pPT104-mediated putrescine uptake by 99.5 percent on the same potABCD operon recovered by LOF lesions in this ALE. Two additional primary references that would strengthen this passage are Kao and Hassan (1985), who measured active [^{14}C]paraquat uptake and characterized a paraquat-tolerant mutant with altered uptake kinetics, and Minton, Tabor, and Tabor (1990), who showed that paraquat toxicity is increased more than 10-fold in E. coli strains defective in spermidine biosynthesis and proposed competition between spermidine and paraquat for intracellular polyanionic binding sites. Neither is in the current bibliography. Fujita et al. (2012) is an Arabidopsis LAT/APC superfamily paper whose structural analogy is to AdiC, a bacterial amino-acid antiporter; LAT/APC and potABCD/ABC are different transporter superfamilies, so it is not the most appropriate structural anchor for a bacterial ABC importer.

The emrE finding directly parallels a result in the authors' earlier paraquat ALE. Rychel et al. (2023), which shares senior authorship (Anand, Yang) with the present manuscript, reported that two of three first-generation paraquat-evolved lineages (1_0 and 2_0) acquired approximately 42-fold amplification in the emrE region (their Figure 3A), and that the third lineage (3_0) and its descendants acquired a 9 bp insertion 39 bp upstream of emrE consistent with an IS1-mediated event. The Patil HPE IS1 insertion at +21/-39 in the renD-emrE intergenic region appears to be a convergent recovery of the 3_0-class mechanism. Rychel et al. (2023) is already cited at this point in the Results (the [10,23,24] cluster) for the general mechanism that IS insertions can alter downstream gene expression, but the specific emrE-activation precedent is not discussed. A sentence that directly ties the HPE IS1 event to the emrE-activation observation in the authors' earlier paper would make the convergence visible to readers.

Either (a) directly measure intracellular paraquat in WT, WTΔpotD, LPE, HPE, and HPE ΔemrE to test whether entry is reduced and efflux is elevated as predicted, or (b) narrow the Results section 2 claims to describe the LPE outcome as an ALE recovery of the previously characterized paraquat-polyamine uptake interaction (Kao & Hassan, 1985; Kashiwagi et al., 1990; Minton et al., 1990), and present the HPE IS1 insertion as a convergent recovery of the IS1-mediated emrE-activation mechanism first observed in Rychel et al. (2023), lineage 3_0.

Referee cross-comment: I agree that the authors should incorporate more of the literature to strengthen the interpretation that LPE strains evolved decreased import, and HPE strains evolved increased export. I strongly disagree that intracellular paraquat needs to be directly measured. We have strong genetic and biochemical knowledge of what these genes do, and there are no other compelling hypotheses for the mechanism of resistance for either LPE or HPE strains.

Authors' response: We have made suitable modifications to the presentation of the results to include details of appropriate references.

2. Two targeted-knockout controls are missing

WTΔemrE baseline paraquat sensitivity is not tested. The ΔemrE experiment is done only in the evolved HPE background (Figure 3C). The manuscript's own data show that the IS1 insertion elevates emrE transcript in HPE relative to WT (Figure 3B). emrE was originally identified as a methyl-viologen antiporter through gain-of-function experiments in which overexpression confers paraquat resistance (Yerushalmi et al., 1995; Morimyo et al., 1992). Two interpretations of the Figure 3C result are therefore compatible with the data: loss of the IS1-driven overexpression state, or loss of emrE function more generally. Adding a WTΔemrE growth curve at 100 μM paraquat alongside the HPE and HPEΔemrE curves would distinguish these directly.

Polyamine pool in ΔpotD is not measured. Minton et al. (1990) established that spermidine specifically (not putrescine) protects E. coli against paraquat toxicity: 10 μM spermidine abolished paraquat growth inhibition in polyamine-deficient strains, whereas putrescine required 1500 μM, and they proposed competition between spermidine and paraquat for polyanionic binding sites (DNA, ribosomes) rather than ROS scavenging. ΔpotD in this work may therefore affect paraquat tolerance through reduced paraquat entry, through altered intracellular spermidine, or through both, and these three scenarios are not equivalent.

Either add WTΔemrE growth curves at 0, 10, and 100 μM paraquat and measure intracellular spermidine and putrescine in WT, ΔpotD, and ΔpotDΔpotF, or note these uncontrolled variables explicitly in the Discussion and soften the mechanistic attribution for both the potD and the emrE lines.

Referee cross-comment: The reviewer proposed testing growth of WT delta-emrE strains on 100 uM paraquat, but the WT strain doesn't grow at all under those conditions (Fig 2C), so that would not work. I also disagree with measuring polyamine levels. The cells are being grown on minimal media, which does not contain any spermidine, while the reference the reviewer cited involved supplementation with 10 uM. Cells in minimal media are not importing spermidine, and the most parsimonious explanation is that reduced paraquat import (based on the literature) is responsible for the evolved phenotype.

Authors' response: We have data for WTΔ*emrE* growth curves at 0, 10, and 100 μM paraquat. As anticipated by the cross-commenting referee, WTΔ*emrE* fails to grow at 10 μM and 100 μM paraquat (Review Figure 1). We have made necessary adjustments to the mechanistic attribution.

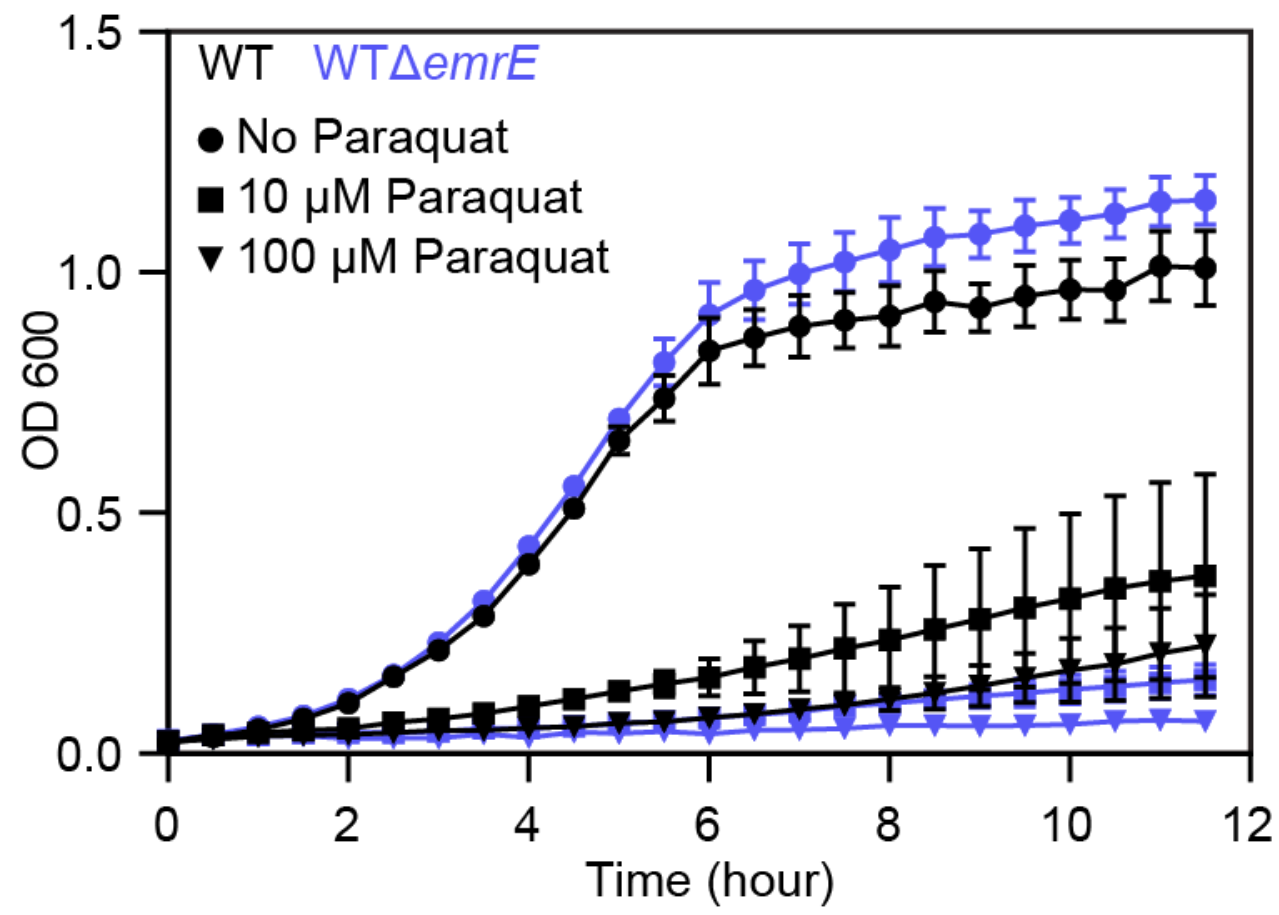

Review Figure 1. Effect of *emrE* gene deletion on the growth profile of the WT strain in the presence of 10 μM and 100 μM paraquat. The growth curve shows a mean of three biological replicates (with three technical replicates each), and the error bars show the standard error of the mean.

3. The StressME cost analysis would benefit from additional parameterization

The StressME extension is useful as parameterized cost accounting. As a genome-scale prediction it has four issues; reframing Figure 5 as parameterized cost accounting would resolve them.

3a. frac_red does not enter the cost calculation. Equation 6 imposes v_import = v_reduction + v_export − v_oxidation under the steady-state assumption v_reduction = v_oxidation. This gives v_export = v_import identically, so PMF cost = 2·v_import and ATP cost = v_import regardless of frac_red. The optimized values of 0.2 (HPE) and 1.0 (LPE) cannot affect the cost bars in Figure 5B. The split-ratio description in Methods should be corrected, or the model reformulated so PQ1OX carries flux and ROS burden varies with frac_red.
3b. Vmax and Km are not identifiable from two dose points. Two paraquat concentrations (10 μM and 100 μM) cannot separately constrain a two-parameter Michaelis-Menten model. When [PQ] is below Km, v_import is approximately (Vmax/Km)·[PQ] and only the ratio is identified. Assigning LPE a higher Km than HPE is therefore equivalent to assigning it a lower cost in the tested range. The cost ranking in Figure 5B follows from the chosen parameter pair rather than from genome-scale inference.
3c. Absolute cost magnitude is not benchmarked. The transport costs in Figure 5B are small relative to ATP maintenance flux in a standard E. coli ME solution. Connecting the six-fold relative cost difference to the competition outcome in Figure 2D would be stronger with a magnitude check: fitting the selection coefficient s from the competition time-course and converting the StressME transport-cost difference into an equivalent growth-rate penalty via the ME proteome-cost model would let the two be compared directly.
3d. PQ1OX carries zero flux in the flux solution. The diagram in Figure 5A depicts a simulated redox-cycling pathway, but the solver does not carry oxidation flux; superoxide burden is imposed through an external bound rather than generated by the redox cycle. Either Figure 5A

should be presented as a cost-accounting schematic, or the model should be run with PQ1OX active and superoxide coupled to v_oxidation.

Either (a) reparameterize with direct [C14]paraquat uptake kinetics across at least five concentrations, activate PQ1OX, and report sensitivity of the cost ranking to Vmax, Km, frac_red, and emrE stoichiometry, or (b) present Figure 5 as a parameterized cost estimate rather than a genome-scale prediction, state which fluxes are fixed inputs versus solver outputs, and temper the Abstract and Conclusion accordingly. Code and data availability are very well handled (StressME v1.2 Docker container and LPE/HPE flux solutions in Dataset S1). A brief supplementary methods note describing what changed from v1.1 to v1.2, and flux solutions for all four lineage-dose combinations, would complete the picture.

Referee cross-comment: The authors should double-check their model to make sure that ATP and PMF costs are correct, but I don't think further parameterization adds value (this is cost-benefit analysis, not enzyme kinetics).

Authors' response: We thank the reviewer for this helpful suggestion and agree that the computational workflow should be presented more explicitly as a parameterized cost-estimation framework built upon the StressME model. Accordingly, we have revised Figure 5 by adding a new schematic that explicitly illustrates the parameterized implementation of paraquat redox cycling in StressME.

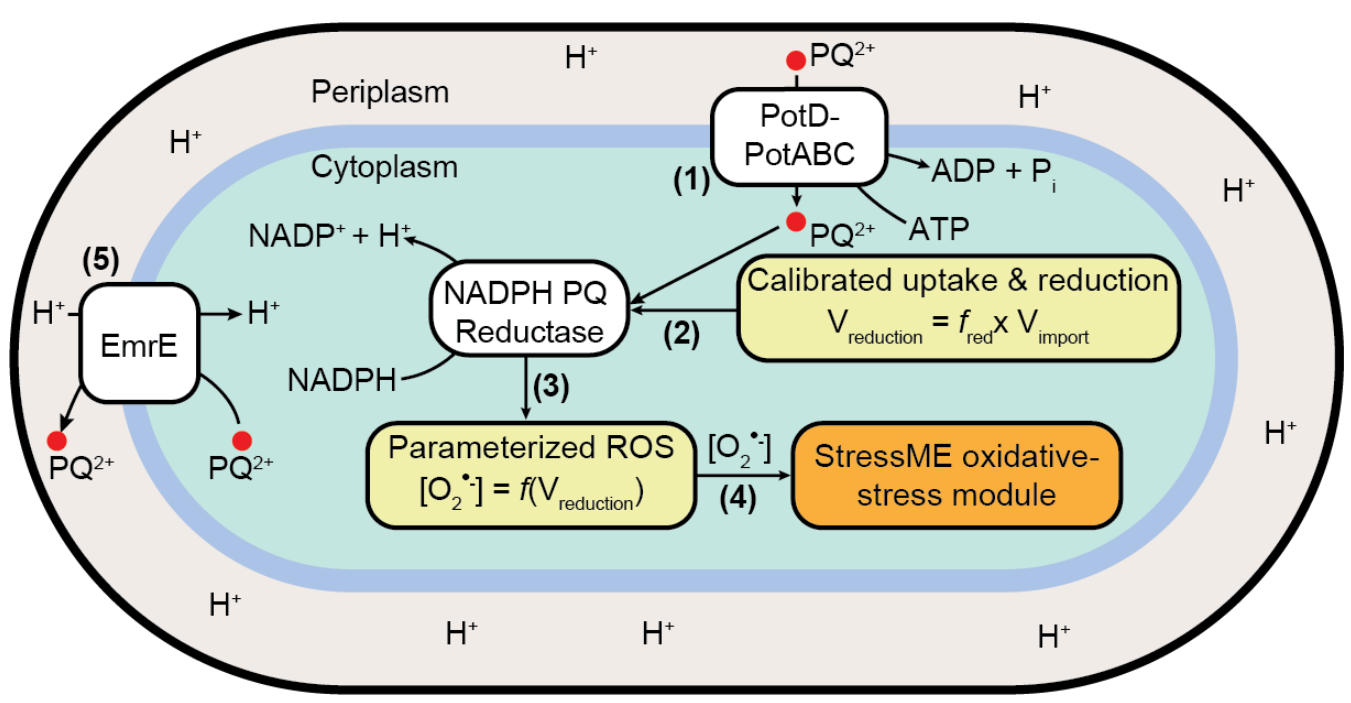

Review figure 2: Parameterized StressME framework

We have also added a supplementary table summarizing the role of each model quantity, distinguishing calibrated parameters, experimental inputs, intermediate calculations, and ME model predictions (Supp. Table 4).

Quantity	Role
Vmax	calibrated parameter
Km	calibrated parameter
fred	calibrated parameter
External PQ	experimental input
v_{import}	computed from Michaelis–Menten kinetics
$v_{reduction}$	computed from f_{red} x v_{import}
Superoxide burden	parameterized from $v_{reduction}$
ATP cost	predicted by StressME
PMF cost	predicted by StressME
Detoxification proteins	predicted by StressME
Repair proteins	predicted by StressME
Growth rate	predicted by StressME

Finally, we have revised the corresponding text throughout the manuscript to consistently describe Figure 5 as a parameterized cost-estimation framework integrated with the StressME model, thereby clarifying which components are calibrated and which cellular responses emerge from the ME model optimization.

We have made suitable modifications to the method description also. We are also providing a tabulated summary of the modifications introduced in StressME v1.2 relative to StressME v1.1 (Supp. Table 3).

Category	StressME v1.1	StressME v1.2
Paraquat metabolites	Not included	pq2_e, pq2_p, pq2_c
Paraquat transport reactions	Not included	PotABCD import, EmrE export, exchange and transport reactions
Protein complexes	Original StressME complexes	Added PotABCD paraquat transporter and EmrE transporter complexes
Expression constraints	Original StressME genes	Added expression coupling for paraquat reductase (b3924) and EmrE (b0543)
Uptake kinetics	Not included	Calibrated Michaelis–Menten uptake (V_{max}, K_m)
Paraquat cycling	Not included	Parameterized $v_{reduction} = f_{red} v_{import}$
ROS input	Native StressME ROS	Paraquat-induced superoxide burden parameterized from $v_{reduction}$
Transport energetics	Native transport costs	Added ATP cost of PotABCD import and PMF cost of EmrE export
Oxidative-stress response	Original StressME	Original StressME driven by paraquat-specific ROS input

We have also included the complete ME solutions for all four lineage–dose combinations (Supplementary Information Dataset S1). For each simulation, the exported results include the flux of every reaction in the ME model together with reaction identifiers and stoichiometric

equations. In addition, a summary output is provided that reports the predicted growth rate, paraquat import, reduction, and export fluxes, the associated ATP and proton motive force (PMF) expenditure, and translation-derived protein synthesis costs for the paraquat reductase and EmrE transporter calculated from the corresponding translation reaction fluxes. These datasets provide the complete numerical outputs presented in the manuscript and were used to generate Figure 5.

Thank you for encouraging us to make our manuscript clearer for a broader readership.

4. The ygfZ interpretation would benefit from reconciling two cited references that report different phenotypes

4a. The Lin et al. (2010) paraquat result differs from how reference 32 is used. Lin et al. tested paraquat explicitly and report that "when paraquat was applied at 1.28 μg per disc, the ΔygfZ strain showed the same resistance as the parental strain, whereas the inhibition zone of the ΔsodA strain increased substantially." They further show that ygfZ overexpression does not suppress paraquat-induced superoxide (their Figure 3C), and their Acknowledgements section states "this is an independent study and has no connection to the recent report by Waller et al." The manuscript cites reference 32 for "ygfZ has been implicated in oxidative stress mitigation," which does not fit the paraquat finding in Lin et al.

4b. Waller et al. (2010) does not experimentally characterize IBA57. The IBA57 claim in Waller et al. is inherited from Gelling et al. (2008). Waller et al. also explicitly favor a "repair of specific enzymes as opposed to de novo synthesis of all Fe/S clusters" framing, so the manuscript's statement that "IBA57 is involved in the assembly of the Fe-S cluster" would be more accurate citing Gelling et al. (2008) directly and saying "repair" rather than "assembly." Mühlenhoff et al. (2022) further report that IBA57 function in mitochondrial [4Fe-4S] maturation is tetrahydrofolate-independent and tentatively extend that conclusion to bacterial ygfZ.

4c. Two cited references contradict each other on the ΔygfZ-paraquat phenotype. Waller et al. (2010) report the deletant "was more sensitive to both stress agents" (their Fig. S3A); Lin et al. (2010) report identical paraquat resistance. This contradiction is not addressed. It also matters for the mechanistic story: an LOF allele producing paraquat tolerance is hard to reconcile with the Waller sensitivity phenotype.

4d. The LH2PE-B intergenic allele (-232/-11) sits in a divergent promoter shared with sdhE. sdhE (b2897) and ygfZ (b2898) are head-to-head, separated by 243 bp. Supp Table 2 annotates this variant as "sdhE←/→ygfZ Intergenic (-232/-11)," acknowledging the divergent-promoter context, but the main-text interpretation groups it with the ygfZ coding mutations. RNA-seq data are deposited (GEO GSE316857), so reporting sdhE and ygfZ transcript ratios in LH2PE-B relative to LH1PE would distinguish whether the intergenic allele acts via sdhE, ygfZ, or both. Rychel et al. (2023) identify the sdhABCD Fe-S clusters as targets of ROS damage in paraquat-evolved strains (their Figure 6F), so sdhE is a plausible target.

Either reconstruct A65V and N215D in WT MG1655 and in LH1PE to test chassis dependence, measure sdhE and ygfZ transcripts in LH2PE-B, or state that LH2PE recovered mutations at the sdhE-ygfZ locus consistent with prior paraquat ALE studies (Rychel et al., 2023; Phaneuf et al., 2024, which compiled 46 ygfZ mutations from ALEdb with L29R, V107E, and T108P as the paraquat-frequent subset clustering near the ethandiol binding sites) and that the specific mechanistic contribution of each allele remains to be determined.

Referee cross-comment: I agree that the authors should try to reconcile references on YgfZ function and their hypotheses, but I don't think that any additional experiments need to be performed on different alleles. The main and important story is the differences in paraquat import/export in cells evolved at different concentrations and the repeatability of said evolution.

Authors' response: Thank you for an elaborate explanation of our existing knowledge regarding YgfZ. We have made appropriate modifications to the manuscript to reconcile the references. We have also updated the references as suggested. We are avoiding detailing the contradiction between Waller et al. (2010) and Lin et al. (2010) for two reasons:
(i) Lin et al.'s have not shown the data,
(ii) We observed something unusual in Waller et al.'s paraquat resistance growth plot. They report no effect of 125 μM PQ on WT *E. coli,* albeit in LB medium. We performed a quick growth profiling and, while less than M9 medium, we observed a growth defect due to paraquat in LB also.

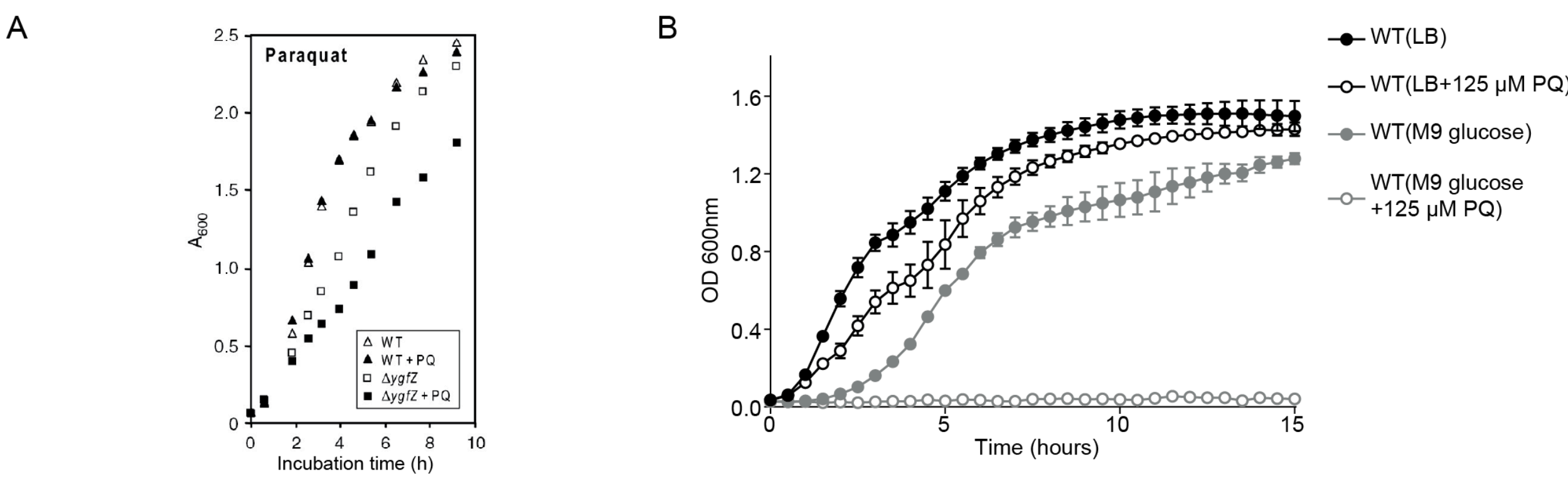

Review Figure 3: (A) Figure S3 taken from Waller et. al (2010) showing growth impact of 125 μM paraquat. (B) Growth profiling of *E. coli* WT strain in LB and M9-Glucose medium with and without 125 μM paraquat. The plot represents two biological replicates with three technical replicates each.

The apparent contradiction between the two studies may be attributable to differences in paraquat concentration or even in the culture medium composition. We hope you will excuse us for not delving into this aspect in the present study.

Technical and editorial points

1. Statistical reporting on emrE expression. The Kruskal-Wallis P = 0.0253 with n = 3 is fragile, and the transcript ratio is carried forward into Equation 8 as the basis for the emrE catalytic-rate inference. Reporting $\log_2$ fold change with 95 percent confidence intervals for each lineage and adding RT-qPCR validation would strengthen this.

Referee cross-comment: I disagree that qPCR is needed--the effect size is large and supported by the literature.

Authors' response: We had performed qPCR analysis for the *emrE* gene before going for transcriptomics. Given the central importance of this expression change, we added this data to the revised manuscript.

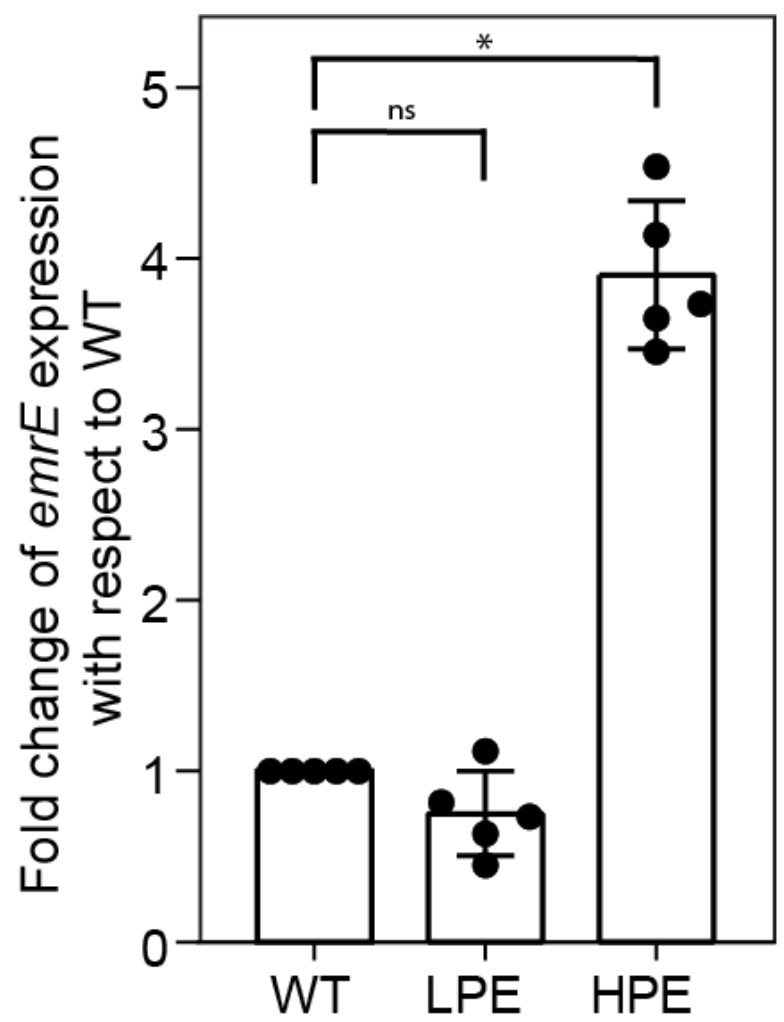

Review Figure 4. Relative expression of the *emrE* gene in LPE and HPE strains with respect to WT. All five individual biological replicates are shown on the plot. Error bars represent standard deviation, and significance was determined using the Kruskal-Wallis test (P-value: * = 0.0308).

2. Competition assay uses an allele-level proxy. WTΔpotD is used as the LPE surrogate and HPE-A as competitor, but WTΔpotD does not carry the growth-promoting rpoB and pyrE-rph mutations that LPE strains acquired. Either repeating with bona fide LPE and HPE lineages or stating this as an allele-level proxy would be appropriate.

Referee cross-comment: True that the WT delta-potD mutant lacks other presumed growth promoting mutations, but that makes the interpretation stronger, since HPE has those mutations and is still outcompeted by the strain.

Authors' response: We added a suitable description in the revised manuscript.

3. IS1 insertion versus amplification in HPE. Given the Rychel et al. (2023) precedent of 42× amplification flanking IS3 elements in two of three lineages, verifying by qPCR or read-depth analysis that the HPE IS1 event is a single-copy insertion rather than a breakpoint of a tandem amplification would rule out the amplification alternative.

Referee cross-comment: It might be good to determine quickly via sequencing of the region or qPCR whether there are amplifications of the region vs an IS1 insertion, but this seems rather discretionary. The main point is that the gene is over-expressed.

Authors' response: We re-examined our genome sequencing data and did not notice an amplification.

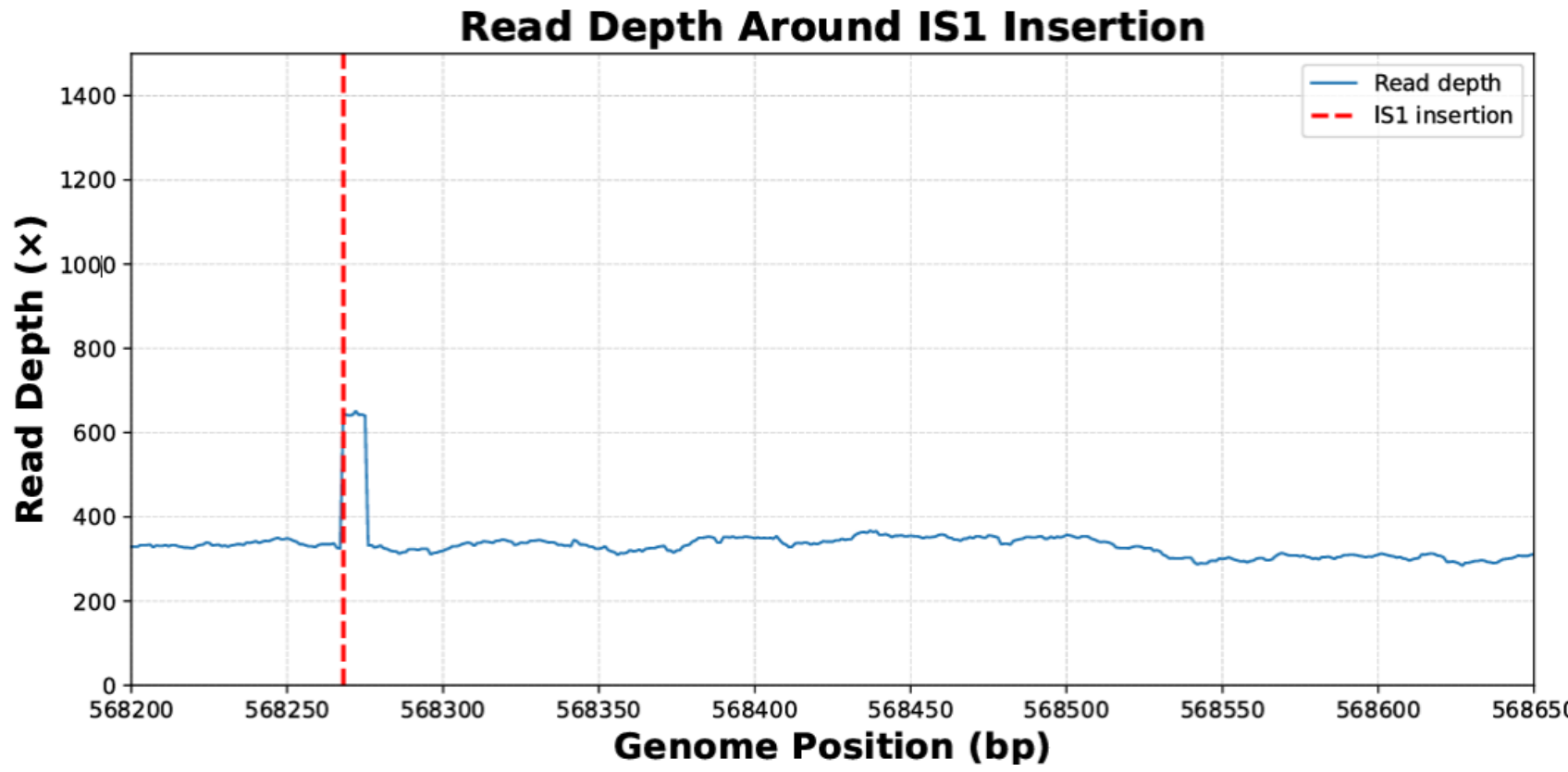

Review Figure 5. Read depth mapping in the LH2PE-B strain surrounding the IS1 insertion site (568268 bp) located in the intergenic region of *renD* and *emrE* genes.

4. The abstract would read more accurately if it acknowledged that the potD-potABC paraquat-uptake connection has a long-standing biochemical literature (Kao & Hassan, 1985; Kashiwagi et al., 1990; Minton et al., 1990) and that IS-mediated emrE activation under paraquat ALE was previously observed by Rychel et al. (2023). Framing the StressME output as a parameterized cost calculation, rather than an emergent genome-scale prediction, would also match what Figure 5 actually delivers.

Authors' response: We have made appropriate modifications to the revised manuscript.

Referee 2

In this manuscript, Patil et al. have run Adaptive Laboratory Evolution (ALE) experiments with E. coli in the presence of two different concentrations of paraquat (10 µM = low dose; 100 µM = high dose), along with high- and low-dose challenges of WT, low paraquat-evolved (LPE) or high paraquat-evolved (HPE) sub-strains, and step-wise evolution. Whole-genome sequencing was used to identify mutations underlying paraquat fitness and RNAseq was used to identify gene expression differences underlying paraquat fitness. Causality is verified for critical fitness genes by targeted mutagenesis. The outcome of this study is a very reasonable model in which adaption to low dose paraquat exposure occurs preferentially by mutation of the relatively low resource-demanding systems that transport paraquat into the cytosol whereas adaptation to high dose paraquat adaptation preferentially uses the higher resource-demanding activation of detoxification and damage-mediating systems. Interestingly, direct evolution in high dose does not appear to modify paraquat import but, along with the detox and damage-repair systems, it did upregulate a multidrug efflux pump that is thought to actively export paraquat.

Overall the experiments seem well designed and rigorous. The authors have made their sequence- and gene expression-data available on a public database; a statement of public availability of the evolved E. coli strains is missing.

Author's response: Thank you for your appreciation of our work.

Concerns.

1. The title, abstract, and much of the body of the paper state that this is a study of oxidative stress tolerance; however, oxidative stress is only indirectly studied. Instead, paraquat stress is studied. Paraquat treatment is an indirect inducer of oxidative stress; the outcomes of paraquat treatment are likely more complicated than simply inducing oxidative stress. This is largely overlooked in the submitted manuscript. Superoxide levels were not measured, only assumed or inferred. There are extended discussions and calculations in the Methods section in support of superoxide production in response to paraquat, but these remain theoretical, not empirical. There is a problem in the redox field and, indeed, extending to other fields that depend on the redox field for mechanistic understanding of redox processes, wherein oxidative stress serves, often without real evidence, as the "usual suspect", implicated for diverse impacts on biological systems wherein actual roles of oxidants are undemonstrated and tenuous. In human health, these implicated effects range from plausible, e.g., mutagenesis, to ridiculous, like aging. Whereas I don't dispute that superoxide radical anion is a byproduct of bacterial paraquat metabolism, it is neither the only product of this metabolism nor is its metabolism without other impacts on the cell. There is no doubt the authors know that it would be correct to have written this paper to precisely say that they are measuring "paraquat tolerance". They chose instead to phrase their title and conclusions on what they interpret from what they did and not what they did. I suspect this is done as a "sales-strategem", jumping on the bandwagon of naming oxidative stress as the usual suspect.

There are two possible resolutions: (1) re-write study study presenting all results and conclusions to precisely implicate paraquat resistance / toxicity and save the speculation about oxidative stress for its appropriate home, within a discrete Discussion section; or (2) rigorously quantitatively validate the roles of superoxide in this system, in the absence of other toxicities or physiological impacts, to fully rule out other influences, and then keep the text "oxidative stress"-focused. Option 2 might, however, be unachievable.

Referee cross-comment: I agree that oxidative stress is too broad, and that the interpretations should be scoped properly as paraquat resistance/tolerance.

Authors' response: We appreciate the reviewer's thoughtful criticism and agree that our original framing was broader than warranted. We acknowledge this oversight and thank the reviewer for highlighting it. Our intention was never to mislead readers or overstate our conclusions. In response, we have revised the manuscript throughout to better align the terminology and interpretations with the experiments performed.

2. Other than one transcriptome heatmap (Fig. 4A), the only empirical measurements in the paper are growth rates. This results in many conclusions being tenuously supported by only indirect evidence. Examples:

a. Superoxide levels were not measured, only inferred. Measuring cellular superoxide and how it changes in each condition would strengthen the study.
b. Paraquat import, metabolism, and export is not measured, only inferred. Indeed, the roles of the PotD-PotABC polyamine-transporter as a paraquat transporter is inferred from the ability of a plant polyamine-transporter to transport paraquat but not directly tested or measured; it's role as the sole paraquat transporter is inferred from (non-impacted) growth characteristics of bacteria with disruption of PotF-PotGHI. Measuring cellular paraquat levels or paraquat metabolic flux in each condition would strengthen the study.
c. Whereas some of the mutations that were mapped would disrupt protein expression, some will make a protein but with a single amino acid change in the PotA ATP-binding domain is predicted to no longer transport paraquat. Transport, however, was not tested. Measuring paraquat transport rates of the WT PotD-PotABC and PotF-PotGHI, as well as the former with the PotA G55D mutation would strengthen the study.

Referee cross-comment: As I mentioned for Reviewer 1, I do not think it's necessary to measure intracellular paraquat or export/import--I think the role of those genes is well supported by the literature, and there are no other compelling hypotheses for the mechanisms of resistance. I think moderating the language (e.g., "these data suggest based on [refs]...") is sufficient.

Authors' response: Our conclusions are based on several studies elucidating the roles of these transporters. We have moderated our statements.

3. I disagree with many of the authors' unnecessarily overly definitive statements, to the point that I find many cases wherein the results don't support the conclusions. As a few examples:

- p. 2, "We performed four separate adaptive laboratory evolutions (ALE) of Escherichia coli under different paraquat concentrations and with varied genetic backgrounds to delineate adaptive strategies to distinct magnitudes of superoxide stress." Problem: The introduction section awkwardly integrates general bacterial environment response with diverse aspects of pathogen-host interactions (not necessarily specific to bacterial pathogens nor are the bacterial responses necessarily exclusive of those in non-pathogenic bacteria). I think the above sentence needs to be preceded with a clarifying clause, such as: "To model potential responses of pathogenic bacteria to these oxidative stresses,..."
- p. 6, "... results ... indicate that PotF-PotGHI does not serve as the primary route for paraquat influx". Problem: PotF-PotGHI paraquat transport function was not measured. The results are consistent with...
- p. 8, "Our findings show that LPE partially decreases paraquat uptake, whereas HPE enhances its export alongside increased ROS detoxification." Problem: Neither decreased

paraquat uptake, enhanced paraquat export, nor increased ROS detoxification were shown, but they are suggested by, or consistent with, the data.
- p. 10, "The simulation showed a difference in PMF and ATP requirements between LPE and HPE strains." Problem: A simulation can allow predictions, not show differences. Neither PMF nor ATP were measured.

Referee cross-comment: I agree with all of this, and have my own comments on the authors' framing. I think the authors would benefit from going through the manuscript carefully to revise these passages and others to be more nuanced.

Authors' response: We have suitably modified the statements.

4. Minor:

a. p. 8, ETS is not defined.
Authors' response: Correction made.

b. p. 10, YgfZ is not defined / described
Authors' response: Correction made.

c. Fig. 5A seems to be missing an electron. NADPH is a 2-electron reactant but only a single radical product is shown.
Authors' response: You are right. The p-orbital of sp^2 nitrogen accepts one electron resulting in generation of paraquat monocation radical. Although NADPH provides a hydride (two electrons), the reductase transfers only one electron to paraquat. The second electron is typically handled through the enzyme's flavin cofactor during subsequent catalytic cycles, so it is not represented as reducing two paraquat molecules in a single reaction cycle. In any case the revised schematic will avoid any stoichiometric confusion.

Referee 3

In this manuscript, Patil et al. used laboratory evolution against low (LPE) and high (HPE) concentrations of paraquat in E. coli and identified two unique mechanisms. For low concentrations of paraquat, LOF mutations in a polyamine transporter were recovered, suggesting that reduced influx is the mechanism of resistance. For high concentrations of paraquat, they observed an IS1 insertion upstream of emrE encoding at multidrug efflux pump, suggesting that paraquat export is necessary for resistance. The HPE mutants were resistant to low concentrations of paraquat, but were outcompeted by LPE cells in competition, suggesting a possible tradeoff. Through modeling, the authors propose that efflux pump upregulation has a higher cost in terms of both ATP costs and proton motive force, which makes sense for decreased import vs increased export. Finally, the authors sequentially evolved the LPE cells on 100 uM and then 200 uM paraquat, where they picked up the IS1 mutation and then another novel mutation in ygfZ that further improved resistance (though the mechanism is unclear).

I think this is an interesting paper, well written and with well-designed experiments. The logic is very clear. I just have one major philosophical disagreement with how the paper is framed. Throughout the paper, the authors frame the paper around "defense strategies" (sometimes hierarchical), and that E. coli "differentially engages modular stress-response programs depending on stress magnitude." This makes it sound like these are deliberate stress response programs, but they are not (unlike for example, the induction of pre-existing ROS defense genes that WT cells have already evolved for tolerating sub-lethal ROS). The cell does not evolve strategies that anticipate becoming a mutant in the future. I think a better framing that still includes the (interesting!) tradeoffs are the differences in evolutionary "paths" towards tolerance, and their predictability (very interesting that the authors obtained the same mutations across evolution experiments, especially the second ALE experiment with the LPE strains). Instead of "Cost adjusted hierarchical defense strategies enables stratified oxidative stress tolerance," something more like "Selection favors cost-ordered mutational paths in a stress-magnitude-dependent manner." I recommend that the authors go through the manuscript and re-frame where appropriate that this addresses which evolutionary paths are favored by selection, including not only tradeoffs but also mutational target size (likely affecting why the same mutations keep arising) and the repeatability of evolution (Stephen Jay Gould's "replaying the tape of life").

Authors' response: We thank you for your encouraging remarks and for suggesting such an appropriate title for our manuscript. We are changing the title to "*Selection favors cost-ordered adaptive mutational paths in a stress-magnitude-dependent manne*r".

Minor critiques:

1) The authors use "flux" control, and it took me a minute to realize that they were talking about influx/efflux of paraquat and not metabolic flux. I think it would help to be more clear about that in places (including the abstract).

Authors' response: We replaced "flux" control with "transport flux" control to make the description precise.

2) In the Methods, I think there should be a few more details about the lab evolution experiments. How many generations for each experiment? How were individual clones selected (just streaked on a plate at the end?), and do the supplemental tables represent every clone sequenced?

Authors' response: We have added more details to the method section.

1st Revision - Editorial Decision 18th Aug 2026

Dear Dr. Anand

Thank you for the submission of your revised manuscript to EMBO reports. We have now received the full set of referee reports that is copied below.

As you can see, the referees find that the study is significantly improved during revision and recommend publication. Before I can accept the manuscript, I need you to address some minor points below:

- Please remove the figures from the manuscript file and place the legends at the end of the manuscript.

- Please rename the "Materials and Methods" section to Methods.

- Please include a statement on blinding in statistical analysis even if no blinding was performed.

- BioRender should be acknowledged at the end of the Methods section in the following way:
Graphics:
(some of the... OR Figure #... OR synopsis) Graphics were created with BioRender.com.

- Please rename the "Conflict of interest" section to Disclosure and Competing Interests Statement.

- Please remove the Reagents and Tools Table from the manuscript file and upload it separately.

- CRediT has replaced the traditional author contributions section because it offers a systematic machine readable author contributions format that allows for more effective research assessment. Please remove the Author Contributions section from the manuscript and use the free text boxes beneath each contributing author's name in our online systems to add specific details on the author's contribution. More information is available in our guide to authors.

- The funders 25P0120 and BT/RLF/Re-entry/70/2020 are missing in the manuscript Acknowledgements section.

- All callouts should be in consecutive order. Fig. 6A is called out before Fig. 5A.

- Please update the file titled (Appendix) Dataset S1 to Dataset EV1. The legend should be removed from the manuscript and provided as a separate tab/sheet in the same excel file.

- Please rename the PDF with tables and one figure to Appendix. Please provide a page number for each item in the table of contents. The correct nomenclature in the file and manuscript should be Appendix Figure S1, Appendix Table S1, etc. The Legend for Appendix Dataset S1 should be removed from the file.

- Thanks for providing the source data (SD). Please upload the SD file folders, which are currently all combined in a single ZIP archive, as one separate archive per each main figure. Source data files need to be saved in a scheme one figure/folder and then uploaded as .zip files. E.g. all the Source data files for figure 1 need to be saved in a single folder and this needs to be zipped and then uploaded as "SD figure 1.zip" file. For EV and/or appendix figures, ZIP together all source data.

- Please provide the specific URLs for datasets (PRJNA1404458 and GSE316857) in the data availability statement.

- EMBO Reports requires that figures with N<5 display individual data points. Please doublecheck that your figures comply with this requirement, particularly Fig. 6B.

- Our production/data editors have asked you to clarify several points in the figure legends (see below). Please incorporate these changes in the manuscript and return the revised file with tracked changes with your final manuscript submission.
- Please note that the exact p values are not provided in the legends of figure 1B.
- Please note that for the figure 4B, p-values and statistical tests are indicated in the legends. However, comparison for the same, ""**"" has not been represented in the figures. Please rectify this in the figures or legends as applicable.
- Please note that the measure of center for the error bars needs to be defined in the legends of figures 3B; 4B.

- Please note that EMBO press papers are accompanied online by:
A) a short (2 sentences) summary of the findings and their significance,
B) 2-5 short bullet points highlighting the key results, and
C) a synopsis image in .jpg or .png format that is exactly 550 pixels wide and 300 pixels high. Please note that the text needs to be legible at the final size. Please upload this information along with your revised manuscript (the text for A and B should be provided in one separate Word file uploaded as Cover Art/Synopsis Image).

We look forward to seeing a final version of your manuscript as soon as possible.

With kind regards,

Kurt Weir
Editor
EMBO Reports

Referee #2:

I find the revised manuscript suitable for acceptance in EMBO Reports.

Referee #3:

I feel that the authors addressed all of my concerns and responded well to those of the other reviewers.

August 21, 2026

Manuscript ID: EMBOR-2026-64056V3

Editor's Comments: **Black Text**
Responses by authors: **Blue Text**

---

1. Please remove the figures from the manuscript file and place the legends at the end of the manuscript.
Authors' response: We have made the suggested changes.

2. Please rename the "Materials and Methods" section to Methods.
Authors' response: We have made the suggested changes.

3. Please include a statement on blinding in statistical analysis even if no blinding was performed.
Authors' response: We have made the suggested changes.

4. BioRender should be acknowledged at the end of the Methods section in the following way:
Graphics:
(some of the... OR Figure #... OR synopsis) Graphics were created with BioRender.com.
Authors' response: We have made the suggested changes.

5. Please rename the "Conflict of interest" section to Disclosure and Competing Interests Statement.
Authors' response: We have made the suggested changes.

6. Please remove the Reagents and Tools Table from the manuscript file and upload it separately.
Authors' response: We have made the suggested changes.

7. CRediT has replaced the traditional author contributions section because it offers a systematic machine readable author contributions format that allows for more effective research assessment. Please remove the Author Contributions section from the manuscript and use the free text boxes beneath each contributing author's name in our online systems to add specific details on the author's contribution. More information is available in our guide to authors.
Authors' response: We have made the suggested changes and updated the contributions in the online system.

8. The funders 25P0120 and BT/RLF/Re-entry/70/2020 are missing in the manuscript Acknowledgements section.
Authors' response: We have made the suggested changes.

9. All callouts should be in consecutive order. Fig. 6A is called out before Fig. 5A.
Authors' response: We have made the suggested changes.

10. Please update the file titled (Appendix) Dataset S1 to Dataset EV1. The legend should be removed from the manuscript and provided as a separate tab/sheet in the same excel file.
Authors' response: We have made the suggested changes.

11. Please rename the PDF with tables and one figure to Appendix. Please provide a page number for each item in the table of contents. The correct nomenclature in the file and manuscript should be Appendix Figure S1, Appendix Table S1, etc. The Legend for Appendix Dataset S1 should be removed from the file.
Authors' response: We have made the suggested changes.

12. Thanks for providing the source data (SD). Please upload the SD file folders, which are currently all combined in a single ZIP archive, as one separate archive per each main figure. Source data files need to be saved in a scheme one figure/folder and then uploaded as .zip files. E.g. all the Source data files for figure 1 need to be saved in a single folder and this needs to be zipped and then uploaded as "SD figure 1.zip" file. For EV and/or appendix figures, ZIP together all source data.
Authors' response: We have uploaded the source data as suggested.

13. Please provide the specific URLs for datasets (PRJNA1404458 and GSE316857) in the data availability statement.
Authors' response: We have made the suggested changes.

14. EMBO Reports requires that figures with N<5 display individual data points. Please doublecheck that your figures comply with this requirement, particularly Fig. 6B.
Authors' response: We have made the suggested changes.

15. Our production/data editors have asked you to clarify several points in the figure legends (see below). Please incorporate these changes in the manuscript and return the revised file with tracked changes with your final manuscript submission.
A) Please note that the exact p values are not provided in the legends of figure 1B.
Authors' response: For Figure 1B, significance was determined using the Mann-Whitney test on GraphPad Prism. The P-value was determined from the software as ****: P-value < 0.0001. An exact P-value was not produced.

B) Please note that for the figure 4B, p-values and statistical tests are indicated in the legends. However, comparison for the same, ""**"" has not been represented in the figures. Please rectify this in the figures or legends as applicable.
Authors' response: We have made the suggested changes.

C) Please note that the measure of center for the error bars needs to be defined in the legends of figures 3B; 4B.
Authors' response: We have made the suggested changes.

16. Please note that EMBO press papers are accompanied online by:
A) a short (2 sentences) summary of the findings and their significance,
B) 2-5 short bullet points highlighting the key results, and

C) a synopsis image in .jpg or .png format that is exactly 550 pixels wide and 300 pixels high. Please note that the text needs to be legible at the final size. Please upload this information along with your revised manuscript (the text for A and B should be provided in one separate Word file uploaded as Cover Art/Synopsis Image).
Authors' response: We have uploaded the required files.

Dr. Amitesh Anand
Tata Institute of Fundamental Research
Department of Biological Sciences
1 Homi Bhabha Road
Colaba
Mumbai, Maharashtra 400005
India

Dear Dr. Anand,

I am very pleased to accept your manuscript for publication in the next available issue of EMBO reports. Thank you for your contribution to our journal.

Your manuscript will be processed for publication by EMBO Press. It will be copy edited and you will receive page proofs prior to publication. Please note that you will be contacted by Springer Nature Author Services to complete licensing and payment information.

You may qualify for financial assistance for your publication charges - either via a Springer Nature fully open access agreement or an EMBO initiative. Check your eligibility: https://link.springer.com/journal/44319/how-to-publish-with-us

Should you be planning a Press Release on your article, please notify the production team via this form in order to coordinate publication and release dates: https://forms.cloud.microsoft/e/eH4A8JVP6g

If you have any questions, please do not hesitate to contact the Editorial Office. Thank you for your contribution to EMBO Reports.

Yours sincerely,

Esther Schnapp, PhD
Senior Editor
EMBO reports

>>> Please note that it is EMBO Reports policy for the transcript of the editorial process (containing referee reports and your response letter) to be published as an online supplement to each paper. If you do NOT want this, you will need to inform the Editorial Office via email immediately. More information is available here: https://link.springer.com/partners/embo-press/editorial-policies#Peer%20review

EMBO Press Author Checklist

Corresponding Author Name: Amitesh Anand	
Journal Submitted to: EMBO Reports	
Manuscript Number: EMBOR-2026-64056V2-Q	

USEFUL LINKS FOR COMPLETING THIS FORM
The EMBO Journal - Author Guidelines
EMBO Reports - Author Guidelines
Molecular Systems Biology - Author Guidelines
EMBO Molecular Medicine - Author Guidelines

Reporting Checklist for Life Science Articles (updated January
This checklist is adapted from Materials Design Analysis Reporting (MDAR) Checklist for Authors. MDAR establishes a minimum set of requirements in transparent reporting in the life sciences (see Statement of Task: 10.31222/osf.io/9sm4x). Please follow the journal's guidelines in preparing your
Please note that a copy of this checklist will be published alongside your article.

Abridged guidelines for figures
1. Data
The data shown in figures should satisfy the following conditions:
- the data were obtained and processed according to the field's best practice and are presented to reflect the results of the experiments in an accurate and unbiased manner.
- ideally, figure panels should include only measurements that are directly comparable to each other and obtained with the same assay.
- plots include clearly labeled error bars for independent experiments and sample sizes. Unless justified, error bars should not be shown for technical
- if n<5, the individual data points from each experiment should be plotted. Any statistical test employed should be justified.
- Source Data should be included to report the data underlying figures according to the guidelines set out in the authorship guidelines on Data

2. Captions
Each figure caption should contain the following information, for each panel where they are relevant:
- a specification of the experimental system investigated (eg cell line, species name).
- the assay(s) and method(s) used to carry out the reported observations and measurements.
- an explicit mention of the biological and chemical entity(ies) that are being measured.
- an explicit mention of the biological and chemical entity(ies) that are altered/varied/perturbed in a controlled manner.
- the exact sample size (n) for each experimental group/condition, given as a number, not a range;
- a description of the sample collection allowing the reader to understand whether the samples represent technical or biological replicates (including how many animals, litters, cultures, etc.).
- a statement of how many times the experiment shown was independently replicated in the laboratory.
- definitions of statistical methods and measures:
  - common tests, such as t-test (please specify whether paired vs. unpaired), simple χ2 tests, Wilcoxon and Mann-Whitney tests, can be unambiguously identified by name only, but more complex techniques should be described in the methods section;
  - are tests one-sided or two-sided?
  - are there adjustments for multiple comparisons?
  - exact statistical test results, e.g., P values = x but not P values < x;
  - definition of 'center values' as median or average;
  - definition of error bars as s.d. or s.e.m.

Please complete ALL of the questions below.
Select "Not Applicable" only when the requested information is not relevant for your study.

Materials

Newly Created Materials	Information included in the manuscript?	In which section is the information available? (Reagents and Tools Table, Materials and Methods, Figures, Data Availability Section)
New materials and reagents need to be available; do any restrictions apply?	Yes	Data Availability Section

Antibodies	Information included in the manuscript?	In which section is the information available? (Reagents and Tools Table, Materials and Methods, Figures, Data Availability Section)
For **antibodies** provide the following information:<br>- Commercial antibodies: RRID (if possible) or supplier name, catalogue number and or/clone number<br>- Non-commercial: RRID or citation	Not Applicable	

DNA and RNA sequences	Information included in the manuscript?	In which section is the information available? (Reagents and Tools Table, Materials and Methods, Figures, Data Availability Section)
**Short novel DNA or RNA including primers, probes**: provide the sequences.	Yes	Materials and Methods, Data Availability Section

Cell materials	Information included in the manuscript?	In which section is the information available? (Reagents and Tools Table, Materials and Methods, Figures, Data Availability Section)
**Cell lines:** Provide species information, strain. Provide accession number in repository **OR** supplier name, catalog number, clone number, and/**OR** RRID.	Not Applicable	
**Primary cultures:** Provide species, strain, sex of origin, genetic modification status.	Not Applicable	
Report if the cell lines were recently **authenticated** (e.g., by STR profiling) and tested for mycoplasma contamination.	Not Applicable	

Experimental animals	Information included in the manuscript?	In which section is the information available? (Reagents and Tools Table, Materials and Methods, Figures, Data Availability Section)
**Laboratory animals or Model organisms:** Provide species, strain, sex, age, genetic modification status. Provide accession number in repository **OR** supplier name, catalog number, clone number, **OR** RRID.	Not Applicable	
**Animal observed in or captured from the field:** Provide species, sex, and age where possible.	Not Applicable	
Please detail **housing and husbandry conditions**.	Not Applicable	

Plants and microbes	Information included in the manuscript?	In which section is the information available? (Reagents and Tools Table, Materials and Methods, Figures, Data Availability Section)
**Plants:** provide species and strain, ecotype and cultivar where relevant, unique accession number if available, and source (including location for collected wild specimens).	Not Applicable	
**Microbes:** provide species and strain, unique accession number if available, and source.	Yes	Materials and Methods

Human research participants	Information included in the manuscript?	In which section is the information available? (Reagents and Tools Table, Materials and Methods, Figures, Data Availability Section)
If collected and within the bounds of privacy constraints report on age, sex and gender or ethnicity for all study participants.	Not Applicable	

Core facilities	Information included in the manuscript?	In which section is the information available? (Reagents and Tools Table, Materials and Methods, Figures, Data Availability Section)
If your work benefited from core facilities, was their service mentioned in the acknowledgments section?	Yes	Acknowledgement

Design

Study protocol	Information included in the manuscript?	In which section is the information available? (Reagents and Tools Table, Materials and Methods, Figures, Data Availability Section)
If study protocol has been **pre-registered, provide DOI in the manuscript**. For clinical trials, provide the trial registration number **OR** cite DOI.	Not Applicable	
Report the **clinical trial registration number** (at ClinicalTrials.gov or equivalent), where applicable.	Not Applicable	

Laboratory protocol	Information included in the manuscript?	In which section is the information available? (Reagents and Tools Table, Materials and Methods, Figures, Data Availability Section)
Provide DOI OR other citation details if **external detailed step-by-step protocols** are available.	Not Applicable	

Experimental study design and statistics	Information included in the manuscript?	In which section is the information available? (Reagents and Tools Table, Materials and Methods, Figures, Data Availability Section)
Include a statement about **sample size** estimate even if no statistical methods were used.	Yes	Materials and Methods, Figure legends
Were any steps taken to minimize the effects of subjective bias when allocating animals/samples to treatment (e.g. **randomization procedure**)? If yes, have they been described?	Not Applicable	
Include a statement about **blinding** even if no blinding was done.	Not Applicable	
Describe **inclusion/exclusion criteria** if samples or animals were excluded from the analysis. Were the criteria pre-established?<br><br>If sample or data points were omitted from analysis, report if this was due to attrition or intentional exclusion and provide justification.	Not Applicable	
For every figure, are **statistical tests** justified as appropriate? Do the data meet the assumptions of the tests (e.g., normal distribution)? Describe any methods used to assess it. Is there an estimate of variation within each group of data? Is the variance similar between the groups that are being statistically compared?	Yes	Materials and Methods, Figure legends

Sample definition and in-laboratory replication	Information included in the manuscript?	In which section is the information available? (Reagents and Tools Table, Materials and Methods, Figures, Data Availability Section)
In the figure legends: state number of times the experiment was **replicated** in laboratory.	Yes	Figure legends
In the figure legends: define whether data describe **technical or biological replicates**.	Yes	Figure legends

Ethics

Ethics	Information included in the manuscript?	In which section is the information available? (Reagents and Tools Table, Materials and Methods, Figures, Data Availability Section)
Studies involving **human participants**: State details of **authority granting ethics approval** (IRB or equivalent committee(s), provide reference number for approval.	Not Applicable	
Studies involving **human participants**: Include a statement confirming that **informed consent** was obtained from all subjects and that the experiments conformed to the principles set out in the WMA Declaration of Helsinki and the Department of Health and Human Services Belmont Report.	Not Applicable	
Studies involving **human participants:** For publication of **patient photos**, include a statement confirming that consent to publish was obtained.	Not Applicable	
Studies involving experimental **animals**: State details of **authority granting ethics approval** (IRB or equivalent committee(s), provide reference number for approval. Include a statement of compliance with ethical regulations.	Not Applicable	
Studies involving **specimen and field samples:** State if relevant **permits** obtained, provide details of authority approving study; if none were required, explain why.	Not Applicable	

Dual Use Research of Concern (DURC)	Information included in the manuscript?	In which section is the information available? (Reagents and Tools Table, Materials and Methods, Figures, Data Availability Section)
Could your study fall under dual use research restrictions? Please check biosecurity documents and list of **select agents and toxins** (CDC): https://www.selectagents.gov/sat/list.htm	Not Applicable	
If you used a select agent, is the security level of the lab appropriate and reported in the manuscript?	Not Applicable	
If a study is subject to dual use research of concern regulations, is the name of the **authority granting approval and reference number** for the regulatory approval provided in the manuscript?	Not Applicable	

Reporting

The MDAR framework recommends adoption of discipline-specific guidelines, established and endorsed through community initiatives. Journals have their own policy about requiring specific guidelines and recommendations to complement MDAR.

Adherence to community standards	Information included in the manuscript?	In which section is the information available? (Reagents and Tools Table, Materials and Methods, Figures, Data Availability Section)
State if relevant guidelines or checklists (e.g., **ICMJE, MIBBI, ARRIVE, PRISMA**) have been followed or provided.	Not Applicable	
For **tumor marker prognostic studies,** we recommend that you follow the **REMARK** reporting guidelines (see link list at top right). See author guidelines, under 'Reporting Guidelines'. Please confirm you have followed these guidelines.	Not Applicable	
For **phase II and III randomized controlled trials**, please refer to the **CONSORT** flow diagram (see link list at top right) and submit the CONSORT checklist (see link list at top right) with your submission. See author guidelines, under 'Reporting Guidelines'. Please confirm you have submitted this list.	Not Applicable	

Data Availability

Data availability	Information included in the manuscript?	In which section is the information available? (Reagents and Tools Table, Materials and Methods, Figures, Data Availability Section)
Have **primary datasets** been deposited according to the journal's guidelines (see 'Data Deposition' section) and the respective accession numbers provided in the Data Availability Section?	Yes	Data Availability Section
Were **human clinical and genomic datasets** deposited in a public access-controlled repository in accordance to ethical obligations to the patients and to the applicable consent agreement?	Not Applicable	
Are **computational models** that are central and integral to a study available without restrictions in a machine-readable form? Were the relevant accession numbers or links provided?	Yes	Data Availability Section
If publicly available data were reused, provide the respective **data citations in the reference list**.	Not Applicable	
